# Clearance of *Treponema pallidum* from oral and anal sites after treatment of secondary syphilis: results from a multi-centre, prospective, cross-sectional study

**DOI:** 10.1016/j.eclinm.2026.104153

**Published:** 2026-08-17

**Authors:** Janet M. Towns, Janath A. Fernando, Rebecca Wigan, Marcelina Krysiak, David A. Lewis, Rick Varma, Anna McNulty, Deborah A. Williamson, Carole Khaw, Jayne Howard, Kate Potappel, Louise Owen, Caroline Thng, Nathan Ryder, Mitchell Hickson, Eric P.F. Chow, Christopher K. Fairley, Stephen R. Graves, Jason J. Ong, Sarah Huffam, Shivani Pasricha, Marcus Y. Chen

**Affiliations:** a Melbourne Sexual Health Centre, Bayside Health, Melbourne, Victoria, Australia; b School of Translational Medicine, Faculty of Medicine, Nursing and Health Sciences, Monash University, Melbourne, Victoria, Australia; c Department of Infectious Diseases at The Peter Doherty Institute for Infection and Immunity, University of Melbourne, Melbourne, Victoria, Australia; d Western Sydney Sexual Health Centre, Western Sydney Local Health District, Parramatta, New South Wales, Australia; e Westmead Clinical School, Faculty of Medicine and Health and Sydney Institute for Infectious Diseases, University of Sydney, Sydney, New South Wales, Australia; f Sydney Sexual Health Centre, Sydney, New South Wales, Australia; g The Kirby Institute, University of New South Wales, Kensington, New South Wales, Australia; h School of Medicine, University of St Andrews, St Andrews, Fife, Scotland; i Adelaide Sexual Health Centre, Infectious Diseases Unit, Royal Adelaide Hospital, Adelaide, South Australia, Australia; j Statewide Sexual Health Service, Hobart, Tasmania, Australia; k Gold Coast Sexual Health, Southport, Queensland, Australia; l Institute for Biomedicine and Glycomics, Griffith University, Gold Coast, Australia; m Pacific Clinic, Newcastle Sexual Health, Hunter New England Local Health District, New South Wales, Australia; n Centre for Epidemiology and Biostatistics, Melbourne School of Population and Global Health, The University of Melbourne, Melbourne, Victoria, Australia; o Australian Rickettsial Reference Laboratory, Barwon Health, Geelong, Victoria, Australia; p Barwon Reproductive and Sexual Health Clinic, University Hospital, Barwon Health, Geelong, Victoria, Australia

**Keywords:** Syphilis, *Treponema pallidum*, Oral, Anal, Clearance

## Abstract

**Background:**

Syphilis is increasing worldwide, and secondary syphilis may represent its most infectious stage. Scarce data exist on *Treponema pallidum* clearance after treatment. We aimed to determine the frequency and load of *T. pallidum* mucosal shedding, and time to clearance following treatment of secondary syphilis.

**Methods:**

Men-who-have-sex-with-men (MSM) and transgender women with secondary syphilis were recruited from Australian clinics between January 27, 2023, and August 19, 2024. Oral, anal or genital lesions were swabbed, and non-lesion samples (blood, oral cavity and anal canal swabs were collected on the day of treatment (baseline). Non-lesion oral cavity and anal canal swabs were repeated 3, 7, 14 and 30 days post-treatment. *T. pallidum* bacterial load was determined by quantitative PCR.

**Findings:**

78 cis-men and one trans-woman, who reported sex with men, were included. At baseline, *T. pallidum* was detected in the participants’ oral cavity, anal canal and blood of 81% (64/79), 70% (55/79) and 24% (19/79), respectively. When detected at oral and anal sites, 22/64 (36%) had oral lesions and 31/55 (59%) had anal lesions, respectively. The median oral and anal bacterial loads decreased significantly from baseline to day 3, [cycle thresholds 35.7–37.7, (1.7–1.2 copies/μL), (p = 0.013), and 30.2–38.2 (3.3–1.0 copies/μL), (p < 0.001), respectively]. *T. pallidum* cleared from 99% (96/97) of non-lesion, paired samples from 58 participants by day 7, 95% confidence interval (94–100%).

**Interpretation:**

During secondary syphilis, most MSM had *T. pallidum* DNA detected in oral and anal mucosa. Bacterial load dropped markedly 3 days post-treatment and cleared in almost all samples by 7 days.

**Funding:**

Australian National Health and Medical Research Council.

**Research in context**

**Evidence before this study**

We searched PubMed for articles of *Treponema pallidum* persistence following treatment of syphilis, published from January 1st, 2010, to August 31, 2025, using the search terms “syphilis”, “*T. pallidum*”, “clearance”, and “after treatment”, without language restrictions. We identified 499 studies, four of which aimed to determine the detection or persistence of *T. pallidum* after treatment for syphilis, in oral/saliva samples, anal swabs, urine, blood, and lesions. Previous studies have also shown that the secondary syphilis stage has the highest frequency of *T. pallidum* DNA detection in oral mucosa, anus and saliva, including among men who have sex with men and people living with HIV. These findings have challenged the traditional model that transmission occurs only from overt genital or mucocutaneous lesions and have raised the possibility of transmission from asymptomatic, non-genital sites. Data on bacterial load and the duration of *T. pallidum* persistence at oral and anal sites following treatment are sparse, leaving an important evidence gap for understanding infectiousness after treatment.

**Added value of this study**

To our knowledge, this is the largest study examining the frequency, bacterial load, and time to clearance of *T. pallidum* from oral and anal sites among men who have sex with men and transgender women with untreated secondary syphilis. Unlike previous studies, which were limited by small sample sizes, heterogeneous sampling methods, mixed syphilis stages, or absence of longitudinal sampling after treatment, this study focused exclusively on secondary syphilis and used a standardised, comprehensive oral cavity sampling method combined with quantitative PCR across multiple time points. We demonstrate that *T. pallidum* DNA is detected in a high proportion of participants from oral and anal sites, often in the absence of visible lesions, and frequently at both these sites within the same individual. We further show that bacterial loads at both oral and anal sites decline rapidly after treatment, with clearance of detectable *T. pallidum* in almost all participants by day 7. These findings provide robust quantitative data defining the short duration of post-treatment persistence of detectable *T. pallidum* at sites relevant to sexual transmission.

**Implications of all the available evidence**

Taken together with existing evidence, our findings indicate that secondary syphilis is characterised by frequent detection of *T. pallidum* DNA at oral and anal mucosal sites, including in the absence of visible lesions. Although the clinical significance of this detection remains uncertain because bacterial viability was not assessed, these findings identify oral and anal mucosal sites as important locations for future studies investigating transmission. The rapid decline in bacterial load and near-complete clearance of detectable *T. pallidum* within 7 days of treatment underscore the critical importance of timely diagnosis and treatment in interrupting onward transmission. These data provide an important evidence base to inform clinical counselling and public health guidance regarding post-treatment infectiousness and the duration of sexual abstinence required to prevent further transmission. Future research should focus on assessing the viability of *T. pallidum* detected at early post-treatment time points and determining whether similar patterns of mucosal shedding and clearance occur in women, heterosexual men, and other populations.

## Introduction

Syphilis, caused by *T. pallidum subspecies pallidum*, remains a global health problem with eight million new infections estimated in adults aged 15–49 years in 2022.^1^ Syphilis disproportionately affects men-who-have-sex-with-men (MSM) and transgender women (TGW).^2^ Syphilis notification rates are rising among heterosexual people in high-income countries, including Australia.^3^^,^^4^ Untreated syphilis causes serious morbidity, including congenital, ocular and neuro-syphilis.

Despite increasing community transmission of syphilis, the mechanisms and timing of sexual transmission of *T. pallidum* remain poorly understood. *T. pallidum* was previously widely assumed to be transmitted from overt primary and secondary mucocutaneous oro-ano-genital lesions. Recent evidence suggests syphilis transmission might also occur from non-genital occult sites or as a result of asymptomatic *T. pallidum* shedding.^5^^,^^6^ The secondary stage has the highest frequency of *T. pallidum* detection in oral mucosa, anus and saliva.^5^, ^6^, ^7^ There are minimal data on the time to *T. pallidum* clearance post-treatment, required to inform patients on the duration of infectiousness and how long abstinence is required to prevent further transmission.^7^^,^^8^

Accordingly, our aim was to determine the frequency of detection, bacterial load and time to clearance from non-genital sites (mouth and anus) in MSM and TGW with secondary syphilis.

## Methods

### Study design

This was a prospective, multisite, cross-sectional study of MSM and TGW with untreated secondary syphilis.

Participants were recruited between January 27, 2023, and August 19, 2024, primarily from Melbourne Sexual Health Centre. Other recruitment sites were Sydney Sexual Health Centre, Sydney; Western Sydney Sexual Health Centre, Parramatta; the Tasmanian Statewide Sexual Health Service, Hobart; Adelaide Sexual Health Clinic, Adelaide, and the Pacific Sexual Health Clinic, Newcastle. Participants were eligible if they were aged 18 years or older, assigned male at birth, identified as MSM or TGW, reported sex with men during the past 12 months, and had laboratory- and clinically-confirmed, secondary syphilis, consistent with both Australian and US Centers for Disease Control definitions.^9^^,^^10^ Secondary syphilis was defined as: “can include localised or diffuse mucocutaneous rash, and/or multiple or multisite lesions to oral and/or anogenital area, e.g. mucous patches, condyloma lata, and alopecia. The primary ulcerative lesion may still be present. The rapid plasma reagin (RPR) is ≥ 1:4”. Syphilis staging was performed by recruiting clinicians and verified by JMT and MYC.

Participants were excluded if secondary syphilis was not confirmed by serology, or if any antibiotics were used within the past month. All participants provided written informed consent.

At the baseline visit, a targeted medical history was obtained including symptoms, previous syphilis infection, sexual behaviour, and current use of HIV pre-exposure prophylaxis (PrEP) using an electronic questionnaire. We did not specifically ask about doxycycline post-exposure prophylaxis use, as such patients were excluded based on recent antibiotic use. All participants were examined for the presence of oral and anal lesions, unless they denied the presence of anal lesions. Proctoscopy was only performed if clinically indicated. All participants were treated with single-dose intramuscular benzathine benzylpenicillin 2.4 mU or, if contraindicated, oral doxycycline 100 mg twice daily for 14 days.

Follow-up visits occurred on days 3, 7, 14, and 30 after treatment. Reimbursement was provided with an AUD$75 gift voucher for baseline and AUD$50 for each follow-up visit.

### Sample collection

At baseline, both lesion swabs and non-lesion study samples were collected. Lesion swabs comprised diagnostic swabs of any oral, anal or genital mucocutaneous lesions were collected from all separate sites in which they appeared, up to 7 days before recruitment, to fully characterise the stage of syphilis. All recruiting sites used local pathology services for diagnostic syphilis and HIV serology, and lesion swab results (Appendix S1, Table S1). Lesion swabs were collected first, followed by non-lesion study samples from oral and anal sites before any routine sexually transmitted infection testing to avoid reducing site *T. pallidum* loads. All laboratory workers processing lesion or non-lesion samples, had information only about the sample types and results, that they were processing, and not to any other study data.

### Study sampling

Non-lesion study samples were an oral cavity swab, anal canal swab and blood sample. All oral cavity and anal canal study samples were collected using a FLOQ Swab (Copan Diagnostics, Murrieta, CA, USA) and obtained from all participants for *T. pallidum* DNA detection, whether local lesions were present or not.

### Oral sampling

The oral cavity was examined by direct inspection of the lips, dorsal and ventral aspects of tongue, both buccal regions, gums and oropharynx, for possible syphilis lesions, which, if present, had a routine diagnostic lesion swab for *T. pallidum* PCR collected. Non-lesion oral cavity swabs were clinician-collected.

Because the optimal method for oral cavity *T. pallidum* detection is unknown, we combined the methods from four studies.^5^, ^6^, ^7^^,^^11^ We used a pre-labelled COPAN red topped tube to sample the entire surface of the oral cavity: dorsal surface of tongue, palate, both buccal mucosal regions, gums, and posterior oropharynx including both tonsils, pressing and rolling the swab during sample collection with firm pressure. After sampling the entire oral cavity surface the swab was left in the mouth for 10 s to collect some additional saliva. The swab was placed in 1 mL DNA/RNA Shield™ tube immediately and vigorously agitated for 20 s. The swab was then removed from the buffer, scraped along the rim of the tube to remove any excess material, then removed and the buffer tube cap replaced. Follow-up non-lesion oral cavity swabs were clinician-collected.

### Anal sampling

The external perianal area was examined, by direct inspection, for possible syphilis lesions, which if present were documented, and a routine diagnostic lesion swab was collected for *T. pallidum* PCR.

Non-lesion anal canal swabs were clinician-collected, at the baseline visit. Follow-up visit non-lesion anal canal swabs were self-collected, unless a clinical examination was indicated. The anal canal swab was collected by inserting the swab 3–4 cm into the anal canal, rotated 5 times, then removed and placed into 1 mL DNA/RNA Shield™ buffer, vigorously agitated for 20 s, and then removed from the buffer. Patient self-collection information sheets were provided to guide the patient-collected samples.

### Genital sampling

A genital examination was not a study requirement but was performed if any lesions were reported, to document and sample primary and secondary lesions (e.g. condylomata lata).

### Blood sampling

A study blood sample was collected at baseline only with a DNA/RNA Shield™ tube containing 6 mL of buffer. Routine diagnostic syphilis and/or HIV serology was also collected.

### Laboratory sample processing

Non-lesion samples (oral cavity, anal canal and blood) were refrigerated for up to 7 days prior to being stored at −80 °C. All study sample processing and testing was performed using an in-house, real-time, quantitative polymerase chain reaction (qPCR) assay targeting the *polA* gene to infer bacterial load, with the limit of detection being four genome copies/mL (Appendix 1, Figure S1). The *polA* gene is a single-copy gene in the *T. pallidum* genome, making it suitable for use across diverse sites where strains with varying repeat gene copy numbers may be present.

Human RNAse P gene detection was performed on all study samples as an internal quality control to verify adequate sample collection and nucleic acid extraction.^12^ Participants with an undetectable human RNAse P result (indicating inadequate sample quality or insufficient cellular material) on a baseline sample, had all samples excluded from the analysis. Any follow up sample with a negative human RNAse P gene control was considered as a missing sample, but other samples from that individual were included in the analysis.

Genomic DNA (gDNA) was extracted using the EZ1&2 Virus Mini Kit v2.0 (QIAGEN, Hilden, Germany) on the EZ1 Advanced XL platform, with a sample volume of 200 μL dissolved into 60 μL elution buffer according to manufacturer's instructions. For each extract, amplification of *polA* and human control RNase P genes were performed in triplicate on the QuantStudioTM 5 instrument (ThermoFisher Scientific, Waltham, Massachusetts, USA) using an in-house quantitative PCR (qPCR) assay (Table S2).^13^ The sequences for each of the TaqMan primers and probes can be found in Table S3. The multiplex qPCR thermocycling conditions were as follows: initial denaturation at 95 °C for 1 min, and 40 PCR cycles of 95 °C for 15 s, 63 °C for 15 s (including data capture), and 72 °C for 10 s. Data were analysed using the QuantStudioTM Design & Analysis Software (v1.5.2).

### Statistical analyses

For categorical variables such as HIV status, reinfections, and site detection we calculated proportions, used the t-distribution method to calculate 95% confidence intervals (CI), and compared using Fisher's exact test. For continuous variables, such as PCR cycle threshold (C_T_) values, we calculated median and interquartile ranges (IQR) of non-lesion oral cavity and anal canal swabs, and used the Wilcoxon-Signed-Ranks-Test to compare anatomical sites by study visit and in the same patient. To determine the frequency and load of *T. pallidum* mucosal shedding, we calculated a required minimum sample size of 50 MSM based on 95% CIs around anticipated proportions of men with oral or anal *T. pallidum* detection, based on oral *T. pallidum* being detected in 40%–88% of participants with secondary syphilis in previous studies.^5^, ^6^, ^7^ P values of less than 0.05 were considered statistically significant. Oral and anal clearance rates were determined from those participants who had non-lesion oral cavity and anal canal samples collected at both baseline and Day 7. Data were analysed using SPSS, version 29.0.0.0.

### Ethics

Study ethical approval was granted by the Alfred Hospital Ethics Committee (462/22) with local research governance approval obtained for each recruitment site. This study was conducted in accordance with the ethical principles of the Declaration of Helsinki.

### Role of funding source

The funder of the study, the Australian National Health and Medical Research Council (NHMRC), had no role in study design, data collection, data analysis, data interpretation, or writing of this report.

## Results

### Participants

**Fig. 1:**
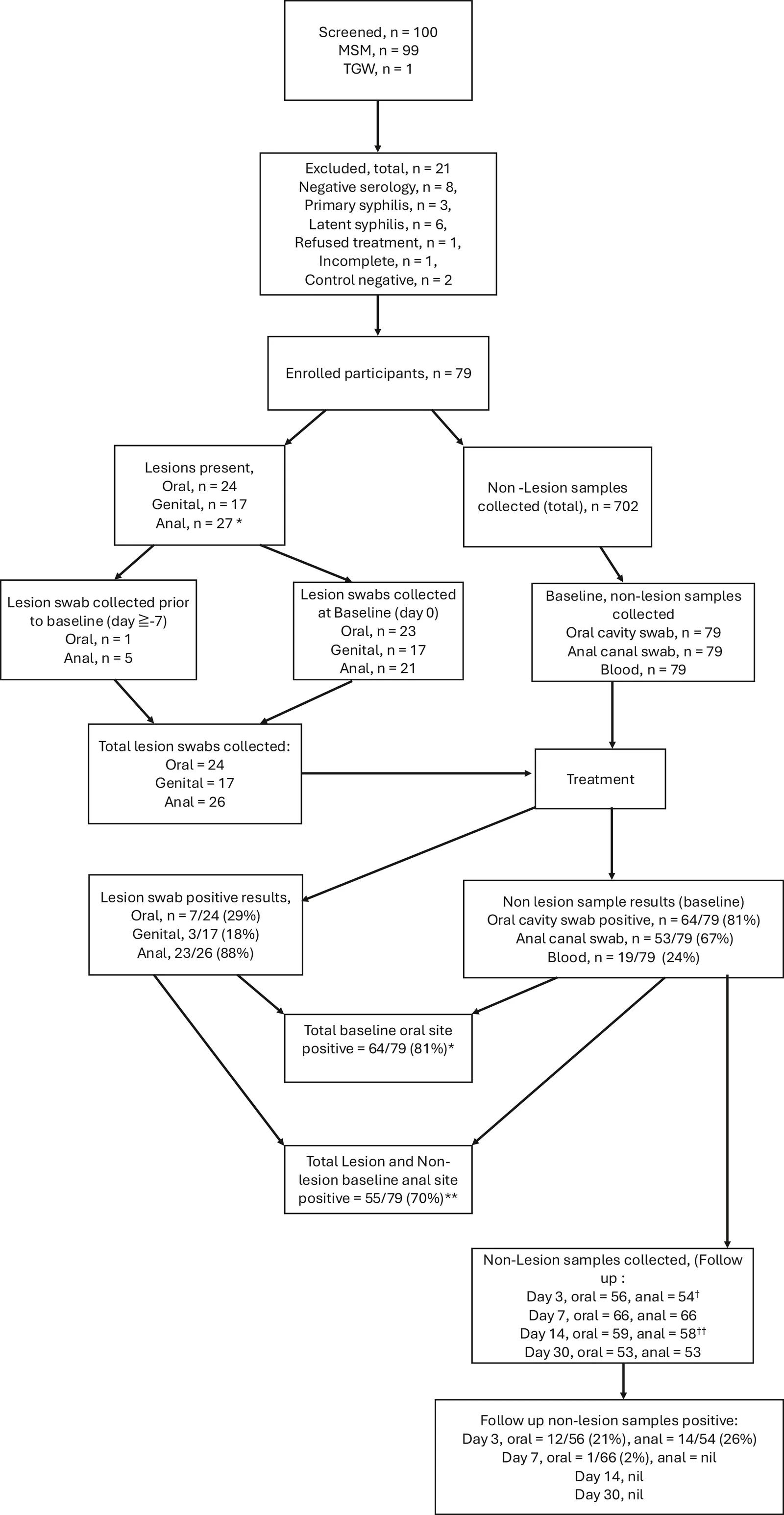
**Flow chart of recruitment, collection of lesion and non-lesion samples, and study results**. Legend: ∗No additional cases of oral detection were contributed from the oral lesion swabs; ∗∗Two additional cases of anal site detection were contributed from the anal lesion swabs; † Two Day 3 anal canal non-lesion samples were excluded from analyses due to a negative control; † † One Day 14 anal swab was excluded from analyses due to a negative control sample.

During the study period 100 participants, including 99 cis-men and one TGW with suspected secondary syphilis were screened for inclusion. Twenty-one were excluded because their serology was negative for *T. pallidum* (n = 8), they had primary (n = 3) or latent (n = 6) syphilis, refused treatment (n = 1), had inadequate sexual history details (n = 1) or the human RNAse P gene was not detected (n = 2) (Fig. 1). Hence, 78 men and one TGW with secondary syphilis were included, Table S4. Among the 16 (20%) participants who were people living with HIV (PLWH), two were diagnosed with HIV at enrolment, 14 (88%) were taking antiretroviral therapy, and 15 (94%) had HIV viral loads less than 50 copies/mL. Twenty-eight (35%) of 79 participants had a syphilis first infection, with no difference in the proportion of reinfections between PLWH and HIV negative participants, (9/16 (56%; 95% CI 29%–84%) versus 19/63 (30%; 95% CI 19%–42%), p = 0.08). Table 1 details the demographic characteristics of the cohort.

**Table 1:** Characteristics of men and transgender women who have sex with men, with secondary syphilis.

Age, years. median (IQR)	32 (27–39)
Country of birth, n (%)	–
Australia	28 (35)
Philippines	6 (8)
India	4 (5)
Brazil	4 (5)
Indonesia	3 (4)
Malaysia	3 (4)
Other	31 (39)
Aboriginal or Torres Strait Islander people, n (%)	2 (3)
Gender, n (%)	–
Cis-Male	78 (99)
TGW	1 (1)
PLWH, n (%)	16 (20)
On ART, n/N (%)	14/16 (88)
Viral load <50 copies/mL^a^, n/N (%)	15/16 (94)
HIV negative, n (%)	63 (80)
Taking PrEP, n/N (%)	29/63 (46)
On-Demand, n/N (%)	12/29 (41)
Daily, n/N (%)	17/29 (59)
Not taking PrEP, n/N (%)	34/63 (54)
Receptive anal sex (in previous 12 months), n (%)	76 (96)
Neurosyphilis^b^	–
Yes, n (%)	2 (3)
No, n (%)	77 (97)
First syphilis infection, n (%)	51 (65)
Syphilis reinfection^c^, n (%)	28 (35)

ART, Anti-retroviral therapy; IQR, Interquartile range, PLWH, persons living with HIV; PrEP, Pre-Exposure Prophylaxis; TGW, Transgender woman.

a One PLWH with a new HIV diagnosis, was taking on-demand PrEP.

b Two participants with secondary syphilis had additional neurological symptoms and were referred for treatment of neurosyphilis.

c Reinfections were either from clinic records or self-reported.

Of the 79 participants, 92% (n = 73) were treated with benzathine penicillin and 8% (n = 6) with doxycycline at baseline. Eight participants had additional antibiotics prescribed at baseline for syndromic management, positive STI results or as contacts of infection: including five prescribed doxycycline, one ceftriaxone, one ceftriaxone plus doxycycline, and one ceftriaxone plus azithromycin. No additional antibiotics were prescribed at the Day 3 time point, but 7 participants had additional antibiotics prescribed at the Day 7 follow up time point. Details of follow up visits are in Table S5. Two participants with neurosyphilis, were treated initially with benzathine penicillin, prior to referral, and subsequently treated with intravenous benzyl penicillin. Two men with rectal symptoms underwent proctoscopic examination.

### Oral detection

**Fig. 2:**
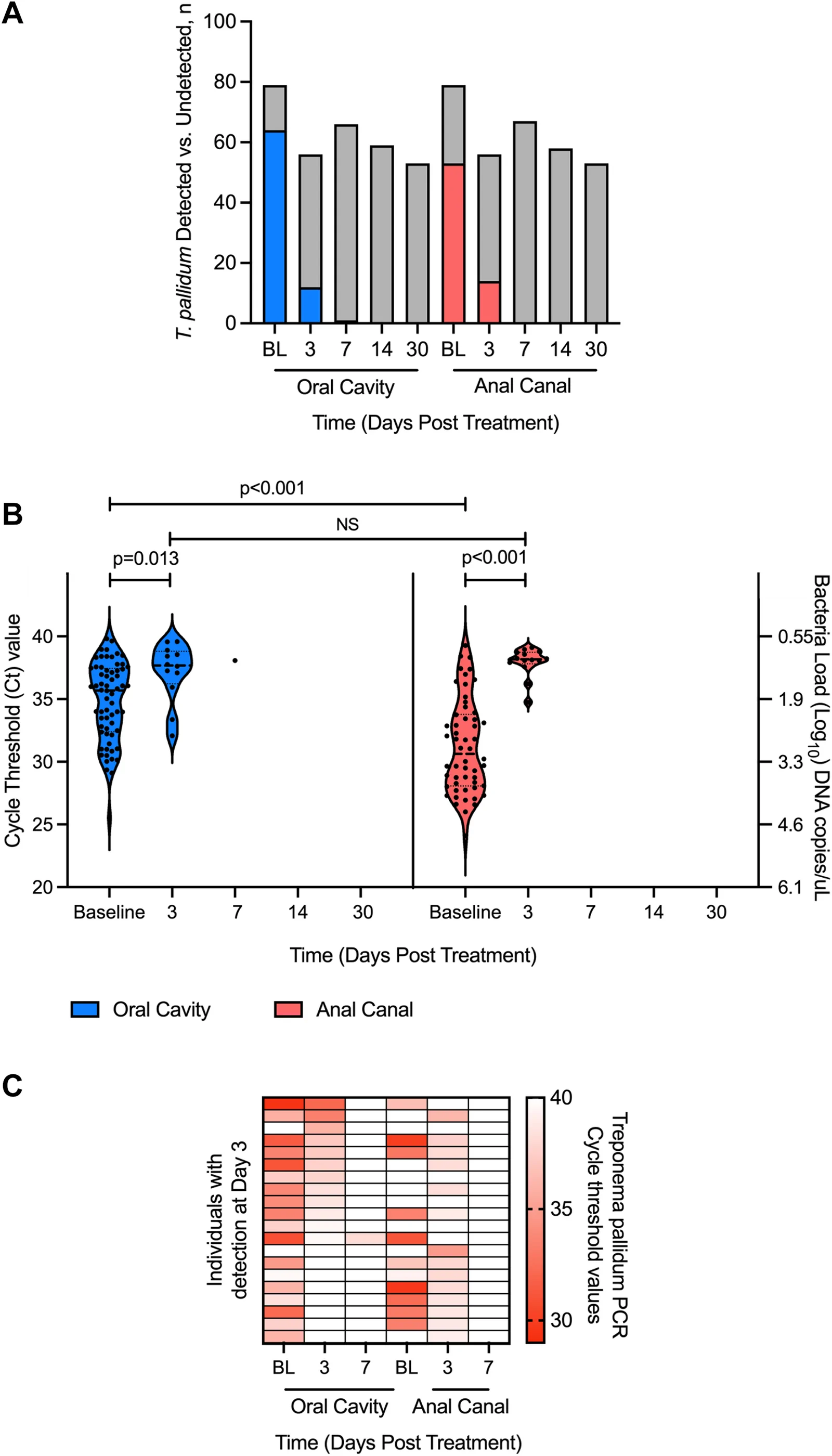
**Non-lesion sample *Treponema pallidum* C_T_ values at baseline and following treatment for syphilis**. Legend: Panel A, detected (red/blue) versus undetected (grey) *T. pallidum* by PCR; Panel B, Violin plot of qPCR cycle threshold (C_T_) values of detected oral and anal samples at baseline (BL), Day 3 and Day 7 post-treatment. Points represent individual samples, medians are shown. Ct values were lower in anal than oral samples at baseline (p < 0.001), but not at Day 3 (p = 0.44); Panel C, Heatmap of *T. pallidum* PCR cycle threshold (Ct) values for paired oral cavity and anal canal samples at baseline (BL), Day 3, and Day 7 post-treatment. Rows represent individual participants with detectable infection at Day 3; columns show sample site and timepoint. Colour intensity reflects Ct values (darker red indicates lower Ct and higher DNA load; lighter shading indicates higher Ct values or no detection).

*T. pallidum* DNA was detected in baseline oral cavity swabs from 64 (81%) of 79 participants (Table 2, Fig. 2A). Of these 64 individuals, 42 (66%) had no oral lesion. Overall, 57/79 (72%) participants had a positive oral cavity swab alone, and 7/79 (9%) were positive in both an oral cavity swab and an oral lesion swab (Fig. 2A). No additional cases of *T. pallidum* were identified from diagnostic oral lesion samples in the absence of a positive oral cavity swab (Fig. 1). There was no significant difference in the frequency of baseline oral detection of *T. pallidum* between PLWH and HIV-negative individuals: 11/16 (69%; 95% CI 43–94) versus 53/63 (84%; 95% CI 75–93), respectively (p = 0.17).

**Table 2:** Clinical and laboratory characteristics of men and transgender women who have sex with men, with secondary syphilis.

All cases, n	(N = 79)
Lesion samples	
Oral lesions present, n (%)	24/79 (30)
Swabbed and PCR positive, n/N (%)	7/24 (29)
Anal, perianal, or rectal lesion(s) present, n (%)	26/79 (33)
Swabbed and PCR positive, n/N (%)	23/26 (89)
Penile or scrotal lesion(s) present, n/N (%)	17/79 (22)
Swabbed and PCR positive, n/N (%)	3/17 (18)
Generalised rash, consistent with secondary syphilis, present, n (%)	70/79 (89)
Any lesion site PCR positive, n (%)	33/79 (42)
Non-lesion samples	–
Oral cavity swab detection, n/N (%)	64/79 (81%)
Anal canal swab detection, n/N (%)	53/79 (67%)
Blood	–
2 or 3 replicates positive, n (%)	7/79 (9)
1 or more replicates positive, n (%)	19/79 (24)
Total baseline lesion or study (non-lesion) sample detection	71/79 (90)
Total oral detection (lesion plus non-lesion), n/N (%)	64/79 (81)
Oral lesion(s) present at site, n/N (%)	22/64 (34)
No oral lesions present at site, n/N (%)	42/64 (66)
Total anal detection (lesion plus non-lesion), n/N (%)	55/79 (70)
Anal lesion(s) present at site, n/N (%)	24/55 (44)
No anal lesions present at site, n/N (%)	31/55 (56)
Participants with non-lesion swabs positive at baseline (total) and day 7 follow up visit, n/N (%)^b^	58/79 (73)
Positive baseline non-lesion oral cavity samples + Day 7 visit, n/N	54/79
Positive baseline non-lesion anal canal swab + Day 7 visit, n/N	43/79
Total positive baseline samples + Day 7 visit, n	97
Proportion samples with undetectable *T. pallidum* at day 7 visit, n/N (%)	96/97 (99)
Treatment	
Benzathine penicillin alone	67/79 (82)
Doxycycline alone	6/79 (8)
Benzathine penicillin plus other baseline antibiotics^c^	8/79 (10)
Serology	–
Treponemal serology reactive (any combination of EIA, CLIA, TPPA, TPHA), n (%)	79 (100)
Non-treponemal serology reactive (either RPR or VDRL titre), n (%)	79 (100)
Median RPR/VDRL titre, median (IQR)^a^	1/64 (1/32–1/128)

a Both test types combined for analysis; CLIA, Chemiluminescent Immunoassay; EIA, Enzyme immunoassay; IQR, interquartile range; PCR, Polymerase Chain reaction; TPHA, *Treponema pallidum* haemagglutination assay; TPPA, *Treponema pallidum* particle agglutination assay; Replicate: number of times sample tested positive out of 3 repeat testings; RPR, Rapid Plasma Reagin; VDRL, Venereal Disease Research Laboratory.

b Non-lesion samples include the study samples of oral cavity swabs, anal canal swabs and blood, and not lesion swabs.

c 8 participants prescribed benzathine penicillin at baseline were prescribed additional antibiotics at this visit, which included five prescribed additional doxycycline, one prescribed ceftriaxone plus doxycycline and one prescribed ceftriaxone plus azithromycin.

Individuals with a first episode of syphilis were more likely to have baseline oral detection, than those with a syphilis reinfection, 46/51 (90%; 95% CI 82–99) versus 18/28 (64%; 95% CI 45–83) (p = 0.007). There was no association between the presence of oral lesions and first infection versus reinfection, 17/51 (33%; 95% CI 10–49) versus 7/28 (25.0%; 95% CI 25–51) respectively, (p = 0.61).

### Anal detection

*T. pallidum* was detected in baseline anal canal swabs from 53 (67%) of 79 participants (Table 2, Fig. 2A). Anal lesions were present in 27/79 (34%) of participants and 23/26 (88%) anal lesion swabs were positive for *T. pallidum* DNA. At the anal site, most baseline detections were identified by non-lesion anal canal swabs (53/79), with lesion swabs contributing only two additional cases, resulting in a total of 55/79 (70%) participants with anal *T. pallidum* detection.

There was no difference in the frequency of anal detection between PLWH and HIV-negative participants: 11/16 (69%; 95% CI 43–94) versus 44/63 (70%; 95% CI 58–81), respectively (p = 1.00). Individuals with a first episode of syphilis were more likely to have baseline anal detection than those with reinfection: 40/51 (78%; 95% CI 67–90) versus 15/28 (50%; 95% CI 34–73), respectively (p = 0.04). There was no difference in the proportion with anal lesions between those with first infection and those with reinfection: 14/51 (28%; 95% CI 17–43) versus 12/28 (43%; 95% CI 26–67), respectively (p = 0.21).

### Multisite detection

Forty-nine (62%) of 79 participants had *T. pallidum* DNA detected at baseline at both oral and anal sites (Fig. 2A). Two had *T. pallidum* detected at baseline at oral, genital and anal sites. None were positive at oral and genital sites only, and one was positive at genital and anal sites only. *T. pallidum* was detected at baseline in 90% (71/79) of participants from one or more anatomical site. Clinical and laboratory characteristics are summarised in Table 2.

### Blood detection

At baseline *T. pallidum* DNA was detected in the blood of 19/79 (24%) participants (Table 2). Blood detection of *T. pallidum* DNA was not significantly different in those with oral detection 15/64 (23%; 95% CI 13–34) versus 4/15 without oral detection (27%; 95% CI 1–52), (p = 0.75), respectively; or those with anal detection 15/55 (27%; 95% CI 15–39) versus without anal detection 4/24 (16.7%; 95% CI 1–33), respectively, (p = 0.40).

There was no difference in *T. pallidum* blood detection in first syphilis infections versus reinfections, 3/28 (11%; 95% CI −2 to 34) versus 16/51 (31%; 95% CI 29–55), (p = 0.05).

### Bacterial load

Baseline blood C_T_ values were significantly higher than for oral or anal sites, indicating lower *T. pallidum* load in blood, median C_T_ 38.4 (0.99 copies/μL), IQR 38.1–38.7, compared with the oral cavity, median C_T_ 35.7 (1.73 copies/μL), IQR 32.5–37.4, (p < 0.001), and anal canal median C_T_ 30.2 (3.25 copies/μL), IQR 28.2–33.6, (p < 0.001), respectively (Fig. 2B).

The baseline C_T_ values for oral cavity swabs were significantly higher than for anal canal swabs, suggesting higher *T. pallidum* loads at the anal site compared to the oral site, (p < 0.001) (Fig. 2B). Day 3 values for oral cavity and anal canal swabs were significantly higher than baseline C_T_ values for oral cavity and anal canal swabs, indicating a decrease in bacterial load at both sites after treatment, median C_T_ 30.2 (3.25 copies/μL) and 38.2 (1.04 copies/μL), (p < 0.001), respectively (Fig. 2B, Table 3). Day 3 C_T_ values for oral cavity swabs and anal canal swabs were not significantly different, (p = 0.075; Fig. 2B).

**Table 3:** Proportion of oral, anal and blood samples with *T. pallidum* detection following treatment for syphilis over time.

	Baseline	Day 3	Percentage difference to baseline	Day 7	Percentage difference to baseline	Day 14	Percentage difference to baseline	Day 30	Percentage difference to baseline
Oral cavity swab	–	–	–	–	–	–	–	–	–
Positive	64	12	57%	1	80%	0	80%	0	80%
Total	79	56		66		59		53	
Proportion (%)	81%	21%		2%		0.0%		0.0%	
Copies/μL	1.7	1.2							
C_T_ value, median (IQR)	35.7 (32.5–37.4)	37.7 (36.2–38.8)	–	NA	–	NA	–	NA	–
Anal canal swab	–	–	–	–	–				
Positive	53	14	42%	0	67%	0	67%	0	67%
Total	79	56		67		58		53	
Proportion (%)	67%	25%		0%		0%		0%	
Copies/μL	3.3	1.0							
C_T_ value, median (IQR)	30.2 (28.2–33.6)	38.2, (37.8–38.7)		NA		NA		NA	
Total collected	158			67					
Total positive	117			1					
Baseline positive AND day 7 sample collected	97								

Note: These data were derived from at least one positive replicate amongst triplicate qPCR reactions.

C_T_, Cycle threshold; NA, not applicable, ND, not done; IQR, interquartile range.

58 participants had study samples collected at both baseline and Day 7, and *T. pallidum* detected in the baseline oral cavity (n = 54) and/or anal canal (n = 43) samples. Clearance by Day 7 was noted in 96/97 (99%) of non-lesion, paired samples from 58 participants (Table 3, Fig. 2C). The longest duration of detection was from one oral cavity swab positive at the second follow-up visit (on day 7) post-treatment (Fig. 2C). This participant had a RPR titre of 1/256 and was treated with oral doxycycline.

## Discussion

In this study, we showed that during secondary syphilis, when most participants had *T. pallidum* detected in oral and anal mucosa, bacterial loads at both sites fell significantly within 3 days after treatment, with clearance in almost all participants by day 7. To our knowledge, this is the largest study to examine the duration of persistence of *T. pallidum* after treatment and the only one focussed on secondary syphilis. The results provide clarity regarding time to clearance of detectable *T. pallidum* following syphilis treatment. We focused on MSM with secondary syphilis because existing evidence indicates that *T. pallidum* is most frequently detected at oral and anal sites in this stage. Our findings highlight the critical role of timely treatment in rapidly reducing detectable *T. pallidum* DNA at oral and anal sites that may be relevant to transmission.

We hypothesise that *T. pallidum* is shed from mucosal sites after systemic dissemination during secondary syphilis. Among MSM and TGW in our study, *T. pallidum* was detected from oral and anal sites in 81% and 70% respectively, and at one or more anatomical sites in 90%, either from a lesion or a site with no observable lesions. *T. pallidum* DNA was detected at both oral and anal sites in 62% of participants, demonstrating frequent detection of *T. pallidum* DNA at multiple anatomical sites within the same individual. The source of shedding in those without visible lesions remains unclear. Our oral cavity sampling method produced over 80% positivity, building on previous research demonstrating high levels of shedding of *T. pallidum* DNA in the secondary stage, and likely benefiting from improved sampling and qPCR-methods.^6^^,^^14^ This suggests *T. pallidum* might be shed from occult lesions, intact mucosa or from saliva.^6^^,^^14^

We sampled multiple sites across several time points following treatment and quantified *T. pallidum* bacterial loads. Our study findings and those of others indicate that oral shedding of *T. pallidum* is also common among PLWH and HIV-negative individuals with syphilis, consistent with previous studies.^5^, ^6^, ^7^ Wang et al. consecutively collected saliva samples from 9 patients at 4-h intervals following treatment, and found the median clearance time for *T. pallidum* DNA in saliva was 64 h.^7^ In a study by Yang et al. of predominantly HIV-positive MSM, 64.5% of men with secondary syphilis had *T. pallidum* detected by oral swab.^11^ Detection of anal *T. pallidum* without lesions has also been reported elsewhere and indicates likely post-dissemination subclinical shedding.^14^, ^15^, ^16^, ^17^ A previous study suggested transmission between MSM is more likely if one person has secondary syphilis, supporting our hypothesis that the secondary stage of syphilis may play an important role in ongoing transmission.^18^

Our study has several limitations. First, while our findings demonstrate clearance of *T. pallidum* by day 7 after treatment, at day 3, when pathogen DNA is still detectable, it remains unclear whether bacteria are viable and therefore transmissible. Despite advances in *T. pallidum* culture methods in recent years, culturing *T. pallidum* from clinical specimens to confirm viability remains challenging. It is possible, that some detected *T. pallidum* DNA is non-viable and non-infectious. Consequently, the presence of detectable DNA should not be interpreted as direct evidence of transmissibility. Second, all participants in this study were MSM or transgender women, which limits generalisability, and therefore the findings cannot be extrapolated to women and heterosexual men. Third, participants in our study did not have routine proctoscopy. Fourth, the lower blood detection rates may be caused by a dilutional effect of the preservative medium. Fifth, the sample size is relatively small, resulting in wide confidence intervals for subgroups, which need to be interpreted cautiously. Although this represents one of the largest prospective studies examining *T. pallidum* clearance following treatment, only 58 participants contributed paired baseline and day 7 samples, limiting our ability to undertake subgroup analyses, including comparisons between treatment regimens. Sixth, we did not collect any post-behavioural data that might affect *T. pallidum* detection or load. Seventh, antibiotic treatments varied between participants, and we do not have adherence data for oral doxycycline usage, limiting analysis of this subgroup.

A strength of our study was our oral sample collection methodology, which was consistently applied to lesions and oral cavity. In previous studies using oral sampling methodology has varied, from combinations of oral lesion swabs,^6^^,^^8^^,^^11^ oropharyngeal swabs,^5^^,^^8^ full or partial oral cavity swabs,^6^^,^^11^ oral rinse samples,^6^ and saliva.^7^ As different oral sampling methods may limit the sensitivity of detection, we combined these methods into a whole oral cavity swab, plus saliva, method. This method appeared optimal for our study, as the oral cavity swab was positive in all cases of oral detection, with and without the presence of lesions.

The association with oral shedding and first infection in our study, but not with reinfections, may indicate the first appearance of partial immunity to *T. pallidum* in patients who have previously been exposed to its antigens. Wang et al. found an association between saliva detection and blood detection between all stages, except primary syphilis, but the proportion of patients in their study with first versus reinfection was not stated.^7^ More studies of reinfected individuals are required to elucidate this further.

In this study we focussed on detection and persistence in the secondary syphilis stage, as previous studies have demonstrated this stage has the highest rates of *T. pallidum* DNA detection. Future research looking to determine the persistence in primary, latent and tertiary syphilis are needed, but these would require different study methodologies.

Studies show increased serological screening of MSM can increase detection of early asymptomatic syphilis, with population-level data suggesting improved screening can prevent progression to secondary syphilis.^19^, ^20^, ^21^, ^22^ Access to, and information on, *T. pallidum* molecular testing should be provided to health-care providers, including primary care physicians, because serology can be negative in individuals with early primary syphilis infection,^23^, ^24^, ^25^ and primary syphilis might be mis-diagnosed as genital herpes.^25^^,^^26^

Oral sex has previously been implicated in the transmission of syphilis,^27^ and emerging biological data demonstrating detection of *T. pallidum* in the oral cavity support this hypothesis.^5^, ^6^, ^7^^,^^11^ The frequent detection of *T. pallidum* provides biological evidence supporting future investigation of anal sexual contact as a route of syphilis transmission. Current guidelines state that a period of 7 days abstinence from sexual activity is required after syphilis treatment.^28^ Although analyses of bacterial load and time to clearance were exploratory, this study represents one of the largest prospective cohorts with frequent longitudinal sampling of *T. pallidum* at mucosal sites, providing detailed insight into early post-treatment dynamics.

To determine the relevance of asymptomatic oral and anal detection to the transmission and control of syphilis, future research should examine the viability of bacteria shed from these sites, bacterial load and clearance from different lesion types, as well as the effect of treatment on the duration of oral and anal shedding of *T. pallidum*, and how these contribute to *T. pallidum* transmission. Further studies in women, heterosexual men and other groups are warranted to determine whether similar patterns of *T. pallidum* shedding occur in other populations.

## Contributors

MYC and JMT conceived and designed the study in consultation with the other authors. Study visits were performed by recruiting clinicians (including RW, JH, KP, DL, MH, RV, AM, SH, LO, NR, CK and CT) at the participating clinics. SP, MK and JF did the laboratory testing. JMT, JF and SP analysed the data. JMT, MYC and SP drafted the manuscript. EPFC and SP provided advice on statistical analysis and reviewed the output with JMT. JA and SP generated the Figures. Recruiting clinicians and JMT assisted with staging of infection, SP oversaw the laboratory work, and SRG contributed to the interpretation of microbiological data. All authors critically reviewed the manuscript for important intellectual content and approved the final version. JMT, RW, JH, KP and SP had access to and verified the underlying study data. All authors had full access to all the data in the study and had final responsibility for the decision to submit for publication.

## Data sharing statement

Clinical and laboratory participant data is included in Supplementary Appendix S2. Other data requests will be considered upon reasonable request.

## Declaration of interests

JAF, MK, DAL, RV, AM, JH, KP, LO, CK, NR, MH, SRG, SH, and SP report no disclosures of interest for this study.

JT is supported by a National Health and Medical Research Council (NHMRC) Partnership Project Grant (APP2003399).

RW is supported by an NHMRC postgraduate scholarship (2030805).

DAW is supported by an Academy of Medical Sciences Professorship (11/1008).

CT is supported by a NHMRC Post-Graduate Fellowship (GNT2022470).

EPFC is supported by an NHMRC Investigator Grant (GNT2033299).

CKF is supported by an NHMRC Investigator Grant (GNT1172900).

JO is supported by an NHMRC Investigator Grant (GNT2042161).

MYC is supported by an NHMRC Investigator Grant 2025840.

## Appendix A Supplementary data

Supplementary Tables

Supplementary Protocol
