# Supplementary material for: Clearance of Treponema pallidum from oral and anal sites after treatment of secondary syphilis: results from a multi-centre, prospective, cross-sectional study
Source: eClinicalMedicine. 2026 Aug 17;99:104153. doi: 10.1016/j.eclinm.2026.104153 (PMC13506794; doi:10.1016/j.eclinm.2026.104153)
Supplement: Supplementary file 1 [file mmc1.pdf]

Clearance of *Treponema pallidum* from oral and anal sites after treatment of secondary syphilis: results from a multi-centre, prospective, cross-sectional study.

Appendix: Sample collection and laboratory methods

Index:

Page 1: Table S1: Syphilis diagnostic tests used at recruiting sites.
Table S2. In-house quantitative PCR (qPCR) assay designed for detection of the *T. pallidum polA* gene; and human RNase P control gene.

Page 2: Table S3. Oligonucleotide sequences of TaqMan sense and antisense primers, and probes used in the multiplex qPCR assay.
Figure S1: Standard curve for limits of detection of *polA* qPCR used for study sample analysis.
Additional PCR methodology.

Page 3 - 9: Table S4. Clinical and laboratory characteristics of whole cohort.

Page 10: Table S5. Follow up visit details.

Table S1: Syphilis diagnostic tests used at each recruiting site.

Recruitment Site	RPR	VDRL	EIA	CLIA	TPPA	TPHA	FTA	*Tp* PCR gene target
**MSHC**	Y*	-		Y**	Y***	-	-	*polA*
**ASHC**	Y	-	Y		Y	-	-	*47kDa*
**SSHC**	Y	-			Y	-	Y	*47kDa*
**TSHS**	Y	-		Y	Y	-	-	*polA*
**WSSH**	-	Y	Y	-	Y	-	Y	*47kDa*
**Pacific**	Y	-		-	Y	-	-	U
**GCSH**			Y			Y	Y	*47kDa*

Key: *, Becton Dickinson, Franklin Lakes, NJ, USA; **, Fujirebio, Tokyo, Japan; ***, LIAISON Treponema screen, DiaSorin, Saluggia, Italy; ASHC, Adelaide Sexual Health Centre; CLIA, Chemiluminescent immunoassay; EIA, Enzyme immunoassay; FTA, Fluorescent treponemal antibody; GCSH, Gold Coast Sexual Health; MSHC, Melbourne Sexual Health Centre; Pacific, Pacific Clinic Hunter New England Area Health Service; RPR, Rapid Plasma Reagin; SSHC, Sydney Sexual Health Centre; *Tp* PCR, *Treponema pallidum* polymerase chain reaction; TPHA, *Treponema pallidum* haemagglutination assay; TPPA, *Treponema pallidum* particle agglutination assay; TSHS, Tasmanian Sexual Health Service; U, unknown; VDRL, Venereal Disease Research Laboratory; WSSH, Western Sydney Sexual Health; Y, Yes.

Table S2. In-house quantitative PCR (qPCR) assay designed for T. pallidum detection of the polA gene, and human RNase P control gene.

Reagent	Required volume per reaction
2x Ex-Taq qPCR Premix (Cat. ID RR390A)	10.0 µL
polA sense primer (10 µM)	0.6 µL
polA antisense primer (10 µM)	0.6 µL
polA FAM probe (10 µM)	0.3 µL
RNase P sense primer (12 µM)	0.5 µL
RNaseP Rev (12 µM)	0.5 µL
RNaseP Texas Red Probe (4 µM)	0.5 µL
Nuclease free water	5.0 µL
gDNA Template	2.0 µL
**Total**	**20 µL**

Table S3. Oligonucleotide sequences of TaqMan sense and antisense primers, and probes used in the multiplex qPCR assay.

Taqman Oligonucleotide	Oligonucleotide Sequence (5' – 3')
polA sense primer	AGGATCCGGCATATGTCCAA
polA antisense primer	GTGAGCGTCTCATCATTCCAAA
polA FAM probe	[FAM]ATGCACGAGCTTCGA[MGBNFQ]
RNase P sense primer	AGATTTGGACCTGCGAGCG
RNase P antisense primer	GAGCGGCTGTCTCCACAAGT
RNase P JUN probe	[Texas Red] TTCTGACCTGAAGGCTCTGCGCG[BHQ2]

Figure S1: Standard curve for limits of detection of *polA* qPCR used for study sample analysis.

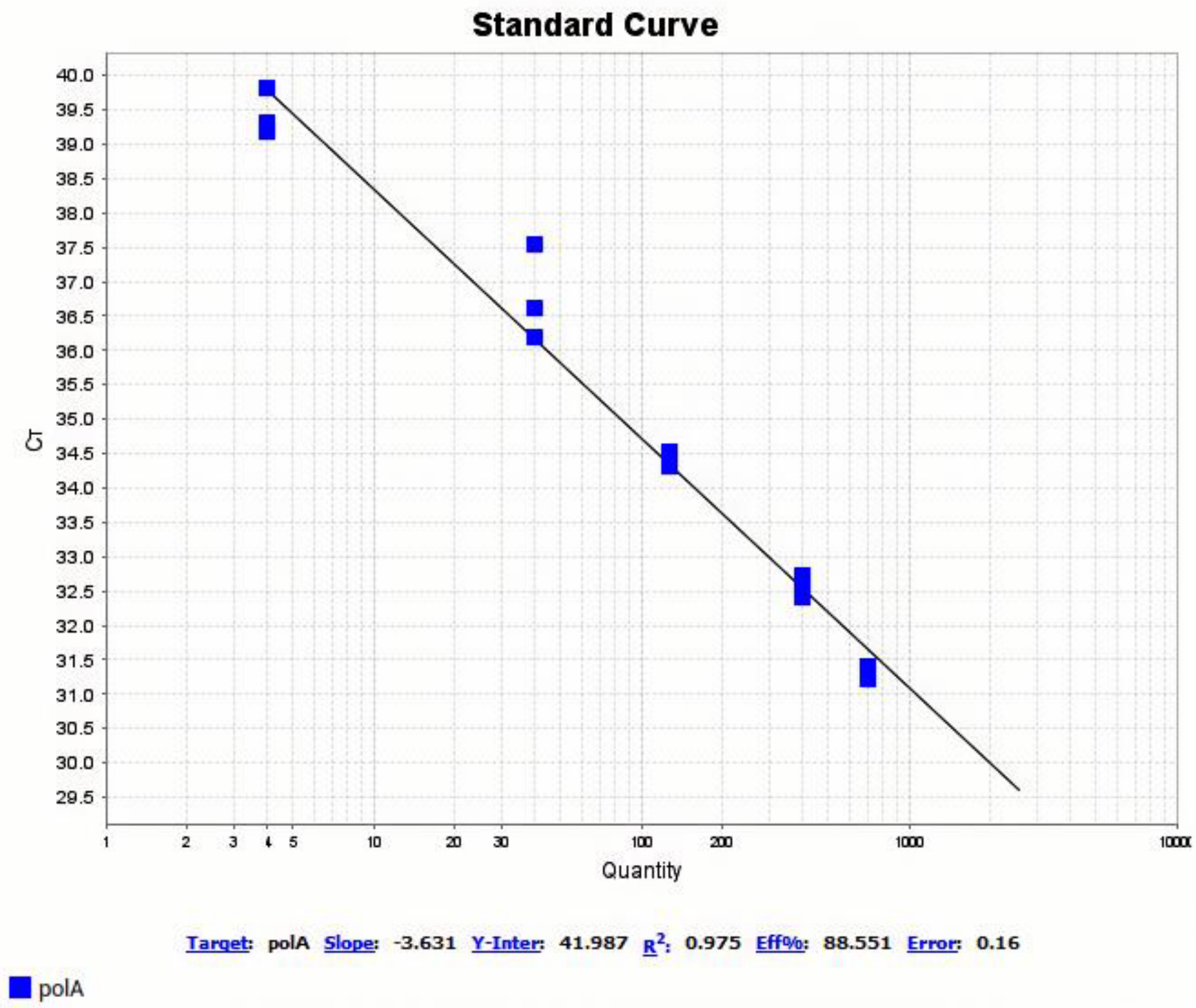

The limit of detection (LOD) was determined following standard methodologies as per previously published work (Low et al. 2023, 2026, Lancet Microbe). Specifically, the LOD was estimated using the Standard Curve in supplementary section 1. To devise the standard curve, commercial quantitated control DNA (Amplirun) with known *Treponema pallidum* copy numbers was diluted to form a set of standards from the neat (i.e., undiluted or 1800 copies/uL) onwards (1:10, 1:100, 1:1000, 1:10000) and the experiments were performed in triplicate. Using the Quantstudio5 qPCR instrument, by specifying that we are using 2 uL per PCR reaction, it will adjust the copies in each well to be copies/uL. Hence, at our limit of detection at around Ct 39.5, our copy number limit of detection ~4 copies. The linear regression curve is made from the QuantStudio5 qPCR instrument itself using the Ct results from the dilution of the standard.

For the standard curve, 2 uL for each reaction well was either the Neat (i.e., undiluted sample), or of the diluted neat sample as described above. For all other PCR reaction, the 2 uL for each reaction was that of the DNA isolate from the clinical sample (i.e., from the 60 uL elution extracted via the EZ1 Advanced XL platform).

Table S4: Clinical and laboratory characteristics of whole cohort.

ID	Repeat Syphilis	HIV-1 status	VL/ cART use	PrEP use	Clinical Description	RPR/VDRL	Oral Cavity swab			Anal canal swab			Blood	Oral lesion	Anal Lesion	Genital Lesion
			(copies/ml)			Titre	Baseline (CT)	FU visit 1 (CT)	FU visit 2 (CT)	Baseline (CT)	FU visit 1 (CT)	FU visit 2 (CT)	(CT)	(CT)	(CT)	(CT)
1	N	-	NA	OD	Genital: rash. Generalised rash.	32	31.0	37.6	ND	27.2	38.2	ND	-	NA	NA	-
2	N	-	NA	OD	Generalised rash.	16	37.0	-	-	33.7	-	-	38.3	NA	NA	NA
3	Y	-	NA	N	Genital: rash. Generalised rash.	8	32.8	ND	ND	33.9	ND	ND	-	NA	NA	NA
4	N	-	NA	D	Anal: condylomata lata.	128	33.7	38.4	-	27.7	38.2	-	-	NA	30	NA
5	Y	-	NA	N	Genital: multiple penile lesions. Generalised rash.	128	35.7	-	-	-	-	-	-	NA	NA	-
6	N	-	NA	D	Generalised rash, tinnitus. NS.	64	31.4	37.1	-	30.0	37.6	-	38.8	NA	NA	NA
7	Y	-	NA	D	Oral: hard palate lesion. Generalised rash.	64	37.6	-	-	33.4	38.8	-	-	-	NA	NA
8	N	-	NA	N	Oral: mucous patches. Generalised rash, abnormal LFT.	32	38.4	-	-	32.3	38.6	-	-	-	NA	NA
9	Y	-	NA	OD	Generalised rash, hair loss	64	37.1	-	-	26.6	-	-	-	NA	NA	NA

ID	Repeat Syphilis	HIV-1 status	VL/ cART use	PrEP use	Clinical Description	RPR/VDRL	Oral Cavity swab			Anal canal swab			Blood	Oral lesion	Anal Lesion	Genital Lesion
			(copies/ml)			Titre	Baseline (CT)	FU visit 1 (CT)	FU visit 2 (CT)	Baseline (CT)	FU visit 1 (CT)	FU visit 2 (CT)	(CT)	(CT)	(CT)	(CT)
10	N	-	NA	N	Oral: mucous patches. Generalised rash, headaches.	64	30.0	ND	-	-	ND	-	-	Y/36	NA	NA
11	Y	-	NA	N	Oral: gum lesion. Generalised rash.	128	36.1	-	-	-	-	-	-	-	NA	NA
12	N	-	NA	N	Oral: mucous patches. Generalised rash	64	37.8	ND	-	-	ND	-	-	-	NA	NA
13	N	-	NA	N	Generalised rash.	64	34.0	38.5	-	-	-	-	38.7	NA	NA	NA
14	N	-	NA	N	Genital: multiple ulcers. Anal: ulcer. Generalised rash.	32	-	-	-	27.3	-	-	-	NA	33	37
15	Y	-	NA	N	Anal: multiple ulcers	256	33.5	38.9	-	33.4	39.0	-	-	NA	36	NA
16	Y	+	279000/N	N	Genital: multiple lesions. Anal: condylomata lata. Generalised rash.	> 512	34.1	ND	-	29.3	ND	-	-	NA	32	-
17	Y	+	<50/Y	NA	Generalised rash.	64	-	ND	-	-	ND	-	-	NA	NA	NA
18	N	+	<50/Y	NA	Generalised rash.	64	34.7	-	-	37.0	37.9	-	-	NA	NA	NA
19	Y	+	<50/Y	NA	Generalised papular rash.	256	38.4	-	-	27.9	-	-	-	NA	34	NA
20	N	-	NA	D	Anal: ulcer, condylomata lata. Generalised rash	128	35.0	-	-	31.8	-	-	-	NA	+	NA
21	N	-	NA	N	Generalised rash.	64	-	ND	ND	28.0	ND	ND	37.9	NA	NA	NA
22	Y	-	NA	D	Generalised maculopapular rash torso/arms, fever.	64	34.0	ND	-	-	ND	-	-	NA	NA	NA
23	N	-	NA	N	Anal: condylomata lata. Generalised rash.	512	33.5	ND	ND	29.7	ND	ND	-	NA	38	NA

ID	Repeat Syphilis	HIV-1 status	VL/ cART use	PrEP use	Clinical Description	RPR/VDRL	Oral Cavity swab			Anal canal swab			Blood	Oral lesion	Anal Lesion	Genital Lesion
			(copies/ml)			Titre	Baseline (CT)	FU visit 1 (CT)	FU visit 2 (CT)	Baseline (CT)	FU visit 1 (CT)	FU visit 2 (CT)	(CT)	(CT)	(CT)	(CT)
25	Y	-	NA	OD	Anal: ulcer. Generalised rash. NS.	64	29.1	ND	-	29.7	ND	-	-	NA	+	NA
26	N	-	NA	N	Oral: condylomata lata, tender erythematous gum lesion. Generalised rash.	128	38.6	-	-	36.4	-	-	38.1	-	NA	NA
27	Y	-	NA	N	Oral: condylomata lata, mucous patches, multiple ulcers. Anal: ulcer.	16	35.7	ND	-	28.9	ND	-	-	-	33	NA
28	Y	+	<50/Y	NA	Generalised rash. Proctitis.	> 512	38.3	-	-	-	-	-	-	NA	NA	NA
29	N	-	NA	N	Oral: ulcer. Anal: multiple lesions. Generalised rash.	V 128	34.0	ND	-	28.7	ND	-	-	-	NA	NA
30	N	-	NA	D	Generalised rash.	32	39.8	-	-	37.4	-	-	37.6	NA	NA	NA
31	Y	-	NA	D	Anal: ulcer. Generalised rash.	64	36.0	-	ND	28.3	39.1	ND	-	NA	+	NA
32	N	-	NA	OD	Oral: mucous patches. Anal: multiple ulcers. Generalised rash. Proctitis.	128	30.1	-	-	27.8	-	-	-	-	27	NA
33	N	-	NA	N	Oral: mucous patches lip and tongue. Genital: healed ulcer. Generalised rash.	V >256	35.4	ND	-	34.4	ND	-	-	-	NA	-
34	N	-	NA	N	Generalised rash.	V 16	32.1	-	-	33.1	38.7	-	-	NA	NA	NA
35	N	-	NA	D	Anal: multiple ulcers. Generalised rash.	V 16	-	ND	-	28.4	ND	-	37.5	NA	+	NA
36	Y	-	NA	D	Oral: multiple ulcers. Anal: multiple ulcers. Generalised rash	V 32	36.8	-	-	31.8	-	-	-	NA	+	NA

ID	Repeat Syphilis	HIV-1 status	VL/ cART use	PrEP use	Clinical Description	RPR/VDRL	Oral Cavity swab			Anal canal swab			Blood	Oral lesion	Anal Lesion	Genital Lesion
			(copies/ml)			Titre	Baseline (CT)	FU visit 1 (CT)	FU visit 2 (CT)	Baseline (CT)	FU visit 1 (CT)	FU visit 2 (CT)	(CT)	(CT)	(CT)	(CT)
37	N	-	NA	N	Generalised rash, abnormal LFTs	128	38.9	-	-	-	H-	-	-	NA	NA	NA
38	N	-	NA	N	Generalised rash, night sweats, fatigue.	32	30.5	-	-	36.2	-	-	37.3	NA	NA	NA
39	Y	-	NA	D	Alopecia	256	-	-	-	-	-	-	-	-	NA	NA
40	N	-	NA	N	Generalised rash.	V >256	32.7	-	-	26.6	-	-	-	NA	NA	NA
41	N	-	NA	D	Oral: lip lesion. Generalised rash.	64	34.0	-	ND	-	-	ND	38.5	-	NA	NA
42	Y	+	<50/Y	NA	Genital: penile papules. Generalised rash.	256	-	-	-	31.7	-	-	-	NA	NA	-
43	N	+	<50/Y	NA	Oral: multiple ulcers. Genital: multiple ulcers. Generalised rash.	512	30.5	-	-	28.8	-	-	-	36	NA	-
44	Y	-	NA	N	Anal: ulcer. Generalised rash.	8	-	-	ND	-	-	ND	-	NA	-	NA
45	N	-	NA	OD	Anal: ulcer. Generalised rash.	V 16	-	-	-	26.9	34.8	-	-	NA	+	NA
46	Y	-	NA	N	Oral: mucous patches. Anal: condylomata lata, multiple blisters.	512	-	-	-	-	-	-	38.3	-	37	NA
47	N	-	NA	N	Oral mucous patches. Generalised rash, abnormal LFT.	256	30.9	39.6	38.1	31.1	-	-	38.4	-	NA	NA
48	Y	+	<50/Y	NA	Generalised rash.	128	-	35.9	-	-	-	-	39.1	NA	NA	NA

ID	Repeat Syphilis	HIV-1 status	VL/ cART use	PrEP use	Clinical Description	RPR/VDRL	Oral Cavity swab			Anal canal swab			Blood	Oral lesion	Anal Lesion	Genital Lesion
			(copies/ml)			Titre	Baseline (CT)	FU visit 1 (CT)	FU visit 2 (CT)	Baseline (CT)	FU visit 1 (CT)	FU visit 2 (CT)	(CT)	(CT)	(CT)	(CT)
49	N	-	NA	N	Oral: condylomata lata. Genital: condylomata lata, multiple ulcers. Alopecia.	128	31.0	-	-	28.7	-	-	-	-	NA	33
50	N	-	NA	OD	Generalised rash.	V 64	36.8	-	-	35.2	-	-	-	NA	NA	NA
51	N	-	NA	N	Generalised rash.	V 32	37.6	39.6	-	-	-	-	-	NA	NA	NA
52	N	-	NA	N	Oral: mucous patches. Genital: mucous patches. Anal: ulcer.	64	29.4	32.1	-	36.6	-	-	-	30.2	-	NA
53	Y	+	<50/Y	NA	Generalised rash torso and feet, headache.	128	39.6	-	-	39.3	38.1	-	38.4	NA	NA	NA
54	N	+	<50/Y	NA	Oral: multiple ulcers. Generalised rash.	128	39.0	-	-	38.4	-	-	-	38.6	NA	NA
55	N	+	<50/Y	NA	Anal: heaing ulcer. Generalised rash.	V 32	37.8	-	-	-	-	-	-	NA	+	NA
56	Y	-	NA	D	Generalised rash torso, limbs, scalp.	32	-	-	-	-	-	-	-	NA	NA	NA
57	N	-	NA	N	Oral: mucous patches. Generalised rash scalp.	32	37.3	-	-	27.0	-	-	-	-	NA	NA
58	N	-	NA	D	Anal: ulcer. Generalised rash.	V 32	32.2	-	-	27.3	-	-	-	NA	+	NA
59	N	-	NA	OD	Anal: ulcer. Generalised rash.	V 128	36.6	ND	-	26.0	ND	-	38.7	NA	+	NA
60	N	-	NA	OD	Generalised rash.	V 64	37.5	ND	-	32.8	ND	-	38.5	NA	NA	NA
61	N	-	NA	D	Anal: ulcer. Generalised rash.	64	31.0	ND	-	30.2	ND	-	39.6	NA	+	NA

ID	Repeat Syphilis	HIV-1 status	VL/ cART use	PrEP use	Clinical Description	RPR/VDRL	Oral Cavity swab			Anal canal swab			Blood	Oral lesion	Anal Lesion	Genital Lesion
			(copies/ml)			Titre	Baseline (CT)	FU visit 1 (CT)	FU visit 2 (CT)	Baseline (CT)	FU visit 1 (CT)	FU visit 2 (CT)	(CT)	(CT)	(CT)	(CT)
62	N	-	NA	N	Generalised rash.	32	36.1	ND	-	29.7	ND	-	-	NA	NA	NA
63	Y	-	NA	N	Generalised rash.	128	-	-	-	-	-	-	-	NA	NA	NA
64	N	-	NA	OD	Generalised rash.	16	37.3	-	-	-	H-	-	38.2	NA	NA	NA
65	N	+	<50/Y	NA	Anal: ulcer, multiple macules. Generalised rash, headaches.	64	35.8	33.4	-	28.1	36.2	-	38.9	NA	34	NA
66	Y	-	NA	N	Oral: multiple ulcers. Genital: plaques. Anal: fissures. Generalised rash.	128	30.2	-	-	32.1	-	-	-	37	35	-
67	N	-	NA	N	Genital: scrotal papules.	64	37.4	37.8	-	-	-	-	-	NA	NA	NA
68	N	-	NA	N	Oral: condylomata lata, multiple ulcers. Genital: mucous patches, multiple lesions. Anal: mutiple ulcers.	64	33.5	37.2	-	32.9	38.1	-	-	32	31	36
69	N	-	NA	N	Genital: ulcer. Generalised rash.	128	36.1	-	-	-	-	-	-	NA	NA	-
70	N	-	NA	OD	Oral: multiple ulcers. Genital: healing ulcer. Generalised rash torso.	64	32.5	-	-	-	-	-	-	33.4	NA	-
71	N	+	<50/N	OD	Generalised rash.	64	36.1	-	ND	29.7	38.1	ND	-	NA	NA	NA
72	Y	+	<50/Y	NA	Generalised rash.	64	-	-	-	-	-	-	-	NA	NA	NA
73	N	+	<50/Y	NA	Generalised rash.	64	38.5	ND	ND	34.8	ND	ND	38.5	NA	NA	NA
74	N	-	NA	N	Oral: lesion upper lip. Generalised rash, patchy alopecia, ear pain.	64	37.5	ND	ND	37.4	ND	ND	-	-	NA	NA

ID	Repeat Syphilis	HIV-1 status	VL/ cART use	PrEP use	Clinical Description	RPR/VDRL	Oral Cavity swab			Anal canal swab			Blood	Oral lesion	Anal Lesion	Genital Lesion
			(copies/ml)			Titre	Baseline (CT)	FU visit 1 (CT)	FU visit 2 (CT)	Baseline (CT)	FU visit 1 (CT)	FU visit 2 (CT)	(CT)	(CT)	(CT)	(CT)
75	N	-	NA	OD	Generalised rash.	V 128	33.1	-	-	29.5	-	-	-	NA	NA	NA
76	Y	-	NA	D	Generalised rash.	4	32.4	ND	ND	-	ND	ND	-	NA	NA	NA
77	N	-	NA	N	Generalised rash.	V 32	-	-	-	-	-	-	-	NA	NA	NA
78	N	-	NA	D	Genital: multiple red macules. Generalised rash.	32	36.2	ND	ND	32.9	ND	ND	-	NA	NA	-
79	Y	+	<50/Y	NA	Genital: multiple ulcers. Generalised rash.	8	-	ND	ND	-	ND	ND	-	NA	NA	-

+, positive; - , negative; >, greater than; <, less than; cART, combination antiretroviral therapy; CT, *T. pallidum* PCR cycle threshold; D, daily; FU, follow up, H-, Human RNAse; HIV, human immunodeficiency virus; LFT, Liver function tests; N, No; NA, not applicable; ND, not done; NR, non-reactive; Ns, neurosyphilis; OD, on demand; PrEP, HIV Pre-exposure prophylaxis; RPR, Rapid plasma reagin; TpPCR, *Treponema pallidum* polymerase chain reaction; U, unknown; V/VDRL, Venereal Disease Research Laboratory; VL, viral load; Y, yes.

Table S5. Follow up visit details.

	Baseline, Day 0	Follow up 1, Day 3	Follow up 2, Day 7	Follow up 3, Day 14	Follow up 4, Day 28	Total
Number attending each visit, n/N (%)	79	56	66	59	53	313
Proportion attending each follow up visit, n/N (%)	-	56/79 (70.9)	66/79 (83.5)	59/79 (74.7)	53/79 (67.1)	234
Collection day post baseline, median (range)	-	Day 3 (2 - 5)	Day 7 (5 - 8)	Day 14 (13 - 17)	Day 29 (27 - 32)	-
Number of follow up visits attended, n						234
None, n/N (%)	-	-	-	-	-	8/79 (10.1)
One, n/N (%)	-	-	-	-	-	3/79 (3.8)
Two, n/N (%)	-	-	-	-	-	8/79 (10.1)
Three, n/N (%)	-	-	-	-	-	25/79 (31.6)
Four, n/N (%)	-	-	-	-	-	35/79 (44.3)
Oral cavity swab collected, n	79	56	66	59	53	-
By Clinician, n/N (%)	79/79 (100)	56/56 (100)	66/66 (100)	59/59 (100)	52/53(98.1)	-
By Patient, n/N (%)	0/79 (0)	0	0	0	1/53 (1.9)	-
Unknown, n	0	0	0	0	0	-
Anal canal swab collected, n	79	54*	66	58*	53	-
By Clinician, n/N (%)	77/79 (97.5)	3/54 (5.6)	2/66 (3.0)	4/58 (6.9)	52/53 (98.1)	-
By Patient, n/N (%)	2/79 (2.5)	50/54 (92.6)	63/66 (95.5)	54/58 (93.1)	1/53 (1.9)	-
Unknown, n/N (%)	0/79 (0)	1/54 (1.9)	1/66 (1.5)	0	0	-
Blood collected, n	79	NA	NA	NA	NA	79

* Three follow-up visit anal canal samples (two at day 3 and one at day 14) were excluded from analyses as Human RNAse P controls were negative. NA, not applicable.
