# Supplementary material for: Clearance of Treponema pallidum from oral and anal sites after treatment of secondary syphilis: results from a multi-centre, prospective, cross-sectional study
Source: eClinicalMedicine. 2026 Aug 17;99:104153. doi: 10.1016/j.eclinm.2026.104153 (PMC13506794; doi:10.1016/j.eclinm.2026.104153)
Supplement: Supplementary file 2 [file mmc2.docx]

**Treponemal shedding, load, viability, and time to clearance in secondary syphilis: implications for transmission.**

**Abstract:**

This document describes the study protocol for determining the prevalence, load, viability, and time to clearance of *Treponema pallidum* shedding in secondary syphilis.

**LIST OF INVESTIGATORS**

**Principle Investigator:**

***Dr Janet M Towns***

Sexual Health Physician

Melbourne Sexual Health Centre

Alfred Health

Phone:	(03) 9341 6260

Fax:	(03) 9341 6269

[jtowns@mshc.org.au](mailto:jtowns@mshc.org.au)

**Co-Investigators**

***Prof Marcus Y Chen***

Melbourne Sexual Health Centre

580 Swanston Street, Carlton Victoria 3053, Australia

Phone:	(03) 9341 6260

Fax:	(03) 9341 6269

Email:	[mchen@mshc.org.au](mailto:mchen@mshc.org.au)

***Professor Christopher K Fairley***

Melbourne Sexual Health Centre

580 Swanston Street, Carlton Victoria 3053, Australia

Phone:	(03) 9341 6200

Fax:	(03) 9341 6269

Email:	[cfairley@mshc.org.au](mailto:cfairley@mshc.org.au)

***Ms Rebecca Wigan***

Melbourne Sexual Health Centre

580 Swanston Street, Carlton Victoria 3053, Australia

Phone:	(03) 9341 6268

Fax:	(03) 9341 6269

Email:	[rwigan@mshc.org.au](mailto:rwigan@mshc.org.au)

**Ms Jayne Howard**

Melbourne Sexual Health Centre

580 Swanston Street, Carlton Victoria 3053, Australia

Phone:	(03) 9341 6268

Fax:	(03) 9341 6269

Email:	[jhoward@mshc.org.au](mailto:jhoward@mshc.org.au)

***A/Prof Eric PF Chow***

Melbourne Sexual Health Centre

580 Swanston Street, Carlton Victoria 3053, Australia

Phone:	(03) 9341 6200

Fax:	(03) 9341 6269

Email:	[echow@mshc.org.au](mailto:echow@mshc.org.au)

***Professor Stephen Graves***

Australian Rickettsial Reference Laboratory

DHRA, Barwon Health, Geelong Hospital

Bellerine Street

Email: [graves.rickettsia@gmail.com](mailto:graves.rickettsia@gmail.com)

***Professor Deborah Williamson***
Director, Victorian Infectious Diseases Reference Laboratory (VIDRL)

Professor of Public Health Microbiology,

Dame Kate Campbell Fellow

NHMRC Investigator Grant Fellow

Department of Infectious Diseases

The University of Melbourne

The Peter Doherty Institute for Infection and Immunity 
792 Elizabeth Street | Melbourne | Victoria | Australia | 3000 
doherty.edu.au

T +61 (0) 3 9342 9636 | M+ 61 (0) 420 874 005
Email: [deborah.williamson@unimelb.edu.au](mailto:deborah.williamson@unimelb.edu.au)

*Professor David Lewis*

Director, Western Sydney Sexual Health Centre

Level 4, Jeffery House, 162 Marsden Street, Parramatta NSW 2150

Tel: +61 (2) 9762 5388, Fax: +61 (2) 9762 5387, Mobile: +61 437 304 035

Email: [david.lewis2@health.nsw.gov.au](mailto:david.lewis2@health.nsw.gov.au)

*Dr Alison Ward*

Head of Unit, Senior Consultant Sexual Health Physician, Adelaide Sexual Health Centre, Royal Adelaide Hospital, CALHN

275 North Terrace

Adelaide SA 5000

Telephone: (08) 7117 2822 Facsimile: (08) 7117 2855

alison.ward3@sa.gov.au

*A/PROF. CAROLE KHAW*

MBBS, DFSRH (UK), FRACGP, PG Cert Pub. Hth. (Sex. Hth.), FAChSHM, AFANZAHPE

275 North Terrace

Adelaide SA 5000

Senior Consultant Sexual Health Physician,

Adelaide Sexual Health Service (formerly Clinic 275),

Telephone: (08) 7117 2822 Facsimile: (08) 7117 2855

email carole.khaw@sa.gov.au

*Dr Caroline Thng*

Senior Medical Officer

Gold Coast Sexual Health Clinic

Southport Health Precinct

Level 4, 16-30 High Street
Southport Q 4215

Phone: (07) 56879200
Fax: (07) 56879212
caroline.thng@health.qld.gov.au

Prof. Louise Owen

MBBS(hons) FRACGP FAChSHM

Sexual Health Physician

Director, Statewide Sexual Health Services

60 Collins St, Hobart T 6166 2672

[louise.owen@ths.tas.gov.au](mailto:louise.owen@ths.tas.gov.au)

Dr Nathan Ryder

Senior Staff Specialist & Clinical Director Sexual Health, Hunter New England Local Health District, NSW, Australia

Tel: +61 2 4016 4536

Mobile/cell: +61 4 3295 4857

Email: nathan.ryder@health.nsw.gov.au

*Professor Anna McNulty*

Director, SSHC

Conjoint Associate Professor, School of Population Health, UNSW

Sydney Sexual Health Centre

Level 3, Nightingale Wing, Sydney/Sydney Eye Hospital, Macquarie St, Sydney 2001

P: (02) 9382 7476 F: (02) 9382 7475

[anna.mcnulty@health.nsw.gov.au](mailto:anna.mcnulty@health.nsw.gov.au)

*Dr Sarah Huffam*

MBBS, FRACP, FAChSHM, MPH&TM, M.Bioethics

Specialist Physician; Infectious Diseases, Sexual Health, General Internal Medicine,

University Hospital Geelong, Barwon Health, Victoria, Australia

Postal address: University Hospital Geelong. PO Box 281 Geelong 3220 Phone: + 61 (0) 44 8861705
Email: sarah.huffam@barwonhealth.org.au

**SUMMARY**

| Study title | Treponemal shedding, load, viability, and time to clearance in secondary syphilis: implications for transmission. |
| --- | --- |
| Protocol version | Version 1.0 |
| Objectives | In any individual ≥ 18 years old, who has been diagnosed with untreated secondary syphilis:   1. To determine the prevalence of subclinical detection of *Treponema pallidum* (*Tp*) from the oral mucosa, vaginal mucosa, anal mucosa, together with the load of *Tp* with subclinical shedding. 2. To determine whether the detection, load, and clearance of *Tp* at mucosal sites, is associated with blood levels of *Tp*. 3. To determine the viability of *Tp* from samples with detected *Tp* 4. To determine whether the above differ between HIV positive and HIV negative men, between men and women, and heterosexual men and men-who have sex with men (MSM), and first or repeat syphilis infections. 5. To determine the prevalence of different *T Pallidum* strains with typing and whole genome sequencing (WGS) and compare between anatomical sites and participant groups. 6. To store study samples for use in subsequent syphilis research. |
| Study design  Sample size | This study is a multicentre cross-sectional design, across multiple sexual health clinics in multiple Australian states.  150-250 individuals will be recruited. Recruitment will continue until at least 50 MSM, 50 women and 50 heterosexual men are recruited.  Minimum of 50 MSM, minimum of 50 heterosexual men, minimum of 50 women. Transgender men and transgender women may also be recruited but there is no set sample size for these sub-groups. |
| Selection Criteria | The inclusion criteria are: any individuals, aged 18 years and above, able to provide informed consent, able to complete the study requirements, with untreated secondary syphilis – with either a clinical or laboratory diagnosis.  The exclusion criteria are: individuals unable to understand English, unwilling to participate in the study, unwilling to have a physical examination, diagnosis of late latent syphilis or syphilis infection of unknown duration, recent (in the last month) antibiotic treatment for syphilis. |
| Study Procedure | Eligible clients with secondary syphilis infection attending Melbourne Sexual Health Centre (MSHC) (and other sites) will be approached to participate in the study.  Written information about the study, syphilis infection and consent form will be provided to the participants.  Participants will be asked to complete the study consent forms  Participants will undergo a clinical examination, documentation and swabbing of any oral or anogenital lesions present.  All participants will provide a saliva sample, oral swab, an anal canal swab and blood sample.  Any individual born with a vagina (women, transgender men, intersex, gender non-binary) will also be asked to provide a vaginal swab.  There is an optional extended study component. Consenting participants will be asked to return for 4 further follow up visits in which swabs and saliva only will be collected.  The samples will be sent to the Victorian Infectious Diseases Reference Laboratory at the Doherty Institute for processing. |
| Analysis | Analysis plan:  Prevalence, load, viability, duration of infection, whole genome sequencing, typing and comparison of *Tp* present in saliva samples, oral swabs, anal canal swabs, vaginal swabs, lesion swabs, and blood samples.  Comparison between HIV positive and negative groups.  Comparison between first and subsequent syphilis infections.  Comparison between women, men who have sex with men, and heterosexual men and other sub-groups. |
| Duration of study | Participants will be recruited until the minimum required sample size of 50 MSM, 50 heterosexual men and 50 women is reached. (150 – 250 total participants) |

**TABLE OF CONTENTS**

1. Background and rationale
2. Study design and methods
a) Study design
b) Number of Participants
c) Duration
3. Participants
a) Inclusion criteria
b) Exclusion criteria
4. List of abbreviations
5. Study outline
a) Study design
b) Study overview
c) Duration of patient participation
d) Study procedure risks
e) Study sites
f) Recruitment
g) Informed consent process
h) History
i) Clinical examination overview
j) Treatment and follow up advice
k) Follow up study procedures
l) Reimbursement
6. Pathology testing
a) Overview
b) Study samples
c) Routine STI testing
d) Specimen Transport
e) Storage of samples
7. Results
   a. Overview
   b. Follow up of routine results
   c. External results
   d. Communication of study results
8. Database collection, management and analysis
9. Statistical considerations
a) Sample size and calculation
b) Analysis plan
10. Confidentiality, storage, and archive of study documents
11. Ethics
12. Covid-19 Contingency plan
13. References
1. **BACKGROUND AND RATIONALE**

Transmission of syphilis in many global settings continues with epidemics internationally but precisely how *Treponema pallidum* (*Tp*) is transmitted between sexual partners remains unclear. There is emerging evidence for the role of asymptomatic transmission from the oral cavity and from the anus.(1-7) These studies all demonstrate asymptomatic detection of *Tp* from the oral cavity, and Towns 2021, Golden and Nieuwenberg and Towns 2022 also demonstrate asymptomatic detection of *Tp* from the anus.(3, 4, 6, 7) Wang, Tantalo and Nieuwenberg also were able to perform blood detection and correlate this to *Tp* detection at other sites.(2, 5, 6) None of these studies demonstrated if the detected *Tp* was viable and therefore definitive attribution to transmission was not possible. There have been no studies, that we are aware of, that have determined detection rates of asymptomatic *Tp* from the vagina.

Yang et al 2015 demonstrated that among HIV positive MSM, 64% of men with secondary syphilis, none of whom had oral ulcers, had *Tp* detected from oral swabs. In early latent syphilis, 28% of HIV positive MSM had detection of oral *Tp* in the absence of oral ulcers.(1)

Wang et al 2020 used saliva samples and demonstrated a 44% detection rate of *Tp* overall, peaking in secondary syphilis (88%).(2) This detection rate in secondary syphilis was the highest of all the studies of oral detection, using nested *Tp* polymerase chain reaction (PCR) and digital droplet PCR, which have high sensitivity.(2)

Golden et al 2019 used pharyngeal swabs analysed with a transcription-mediated amplification *Tp* PCR assay and found an overall oral *Tp* detection rate of 25%, peaking in secondary syphilis at 38%).(3)

Towns et al 2021 and Tantalo et al 2021 both used a combination of oral lesion swabs and either oral rinse or oral cavity swab to determine total detection rates of 24% and 27% respectively, peaking in the secondary stage at 44% and 40% respectively.(4, 5) Both of these studies used standard PCR methodology for detection of *Tp*.(4, 5)

Nieuwenberg et al 2022 used an oropharyngeal swab with PCR methodology and found an overall *Tp* detection rate of 33%, peaking in secondary syphilis at 64%.(6)

Tantalo et al 21, Wang et al 2020 and Nieuwenberg et al 2022 all determined overall blood detection rates of *Tp* at 27%, 30% and 33% respectively.(2, 6)

Towns et al 2022 performed a sub analysis of previously collected data and compared the load of *Tp* between subsets of men and rapid plasma reagin (RPR), a syphilis serological test, levels.(4, 7) They found that the load of *Tp* was greater at the anus than in the oral cavity and that the frequency of detection increased with increasing RPR titres.(7)

The average overall detection rate from the oral cavity of *Tp* overall was 30%, with an average peak in secondary syphilis of 53.5%. The sampling methodology with the highest rates of detection were shown in Wang et al, using saliva samples.(2)

Only two of these studies of oral detection of *Tp* recruited women, and none collected vaginal swabs for detection of *Tp*.(2, 5) Three of the studies of oral detection also systematically collected anal swabs for detection of *Tp*, but these studies were all of men who have sex with men (MSM) only.(3, 4, 6) It is unknown whether females and heterosexual men also shed *Tp* from the anus during secondary syphilis.

None of these 6 studies were able to determine viability or duration of viability of the detected *Tp*, and only 2 attempted to determine the duration of detection of detected *Tp*, with mixed results. We are unaware of any previous studies that have attempted to determine the duration of viability of *Tp* from any body sites. The gold standard for determination of viability of *Tp* involves rabbit infectivity testing (rabbit inoculation with fresh material from lesions), which is costly and time consuming. Our laboratory provider is developing an *in vitro* viability assay, which will be used in this study and obviate the need for sacrificing hundreds of rabbits. We are unaware of any previous studies that have used an *in vitro* viability assay for detection of *Tp*, but it has been proven to be effective for other bacterial species.(8) These previous 6 studies have repeatedly demonstrated a peak of *Tp* detection in secondary syphilis, therefore this proposed study will recruit only individual with clinical or laboratory confirmed secondary syphilis.

In summary, there are several knowledge gaps that will be explored in this proposed study:

- Patterns of *Tp* detection in heterosexual men and women.
- Whether detected *Tp* from asymptomatic sites is viable.
- Duration of *Tp* detection and viability.

2. **STUDY DESIGN AND METHODS**
a) **Study design**

The study is a cross-sectional multi-centre design.

b) **Number of participants**

A minimum of 50 MSM, 50 heterosexual men and 50 women will be recruited. Transgender, intersex, and other individuals with non-binary gender identification may also be recruited.

c) **Duration**

The study will run from 17 October 2022 until 31 December 2025.

3. **PARTICIPANTS**
a) **Inclusion Criteria:** Any individual aged ≥18 years of age with at least one sexual partner in the last 12 months.
i. Any individual with untreated clinically suspected or laboratory confirmed secondary syphilis. Must have rash or lesion(s) suggestive of secondary syphilis infection. May have positive PCR result, positive Dark Ground Microscopy result from a lesion or positive syphilis serology, but these are not necessary at the time of enrolment.
ii. Sufficient English language proficiency to understand the requirements of the study.
iii. Willing and able to complete study procedures
iv. Provision of written informed consent
b) **Exclusion criteria:**
i. Individuals under 18 years of age
ii. Individuals who have received any antibiotic treatment within four weeks prior to enrolment.
iii. Diagnosis of primary syphilis or latent syphilis.
iv. Participants unable to provide informed consent
4. **LIST OF ABBREVIATIONS**

CPMS	Clinical Practice Management software/System

FUN	Follow up nurse

HIV	Human immunodeficiency virus

MSHC	Melbourne Sexual Health Centre

MSM	Men who have sex with men

MDU	Melbourne Diagnostic Unit

MO	Medical officer

PICF	Participant information and consent form

PCR	Polymerase chain reaction

RN	Research Nurse

RPR	Rapid Plasma Reagin

SOS	Shedding of spirochaetes (study)

STI	Sexually Transmitted Infection

*Tp*	*Treponema pallidum*

VIDRL	Victorian Infectious Diseases Reference Laboratory

WGS	Whole genome sequencing

5. **STUDY OUTLINE**
a) **Study design**

This is a multi-centre cross sectional prospective study of untreated secondary syphilis infection.

b) **Study overview**

|  | **All participants** | **Optional Study Procedures** |
| --- | --- | --- |
| **Saliva and oral swabs** | X |  |
| **Anal canal swab** | X |  |
| **Blood sample** | X |  |
| **Vaginal swab*** | X** |  |
| **Additional study visits** |  | X |
| **Photography** |  | X |
| **Reimbursement*** | X |  |

*** Reimbursement will be tiered, according to number of study visits**

**** Individuals with a vagina**

c) **Duration of patient participation**

The duration of participation for this study will be up to 5 visits, over 30 days.

d) **Study procedure risks**

The risks and discomfort to participants is minimal. The collection of swab samples may cause minor discomfort but should not cause any pain. Examination procedures will be the same as for clients not undertaking a study. Participants may be embarrassed due to the nature of the physical examination and sample collection.

The anal canal swabs, vaginal swabs, oral swabs, and blood sampling for this study are similar to that performed for routine sexual health examination and testing. The additional saliva sample should not cause any physical discomfort or risk to the patient. No more than an extra 4 ml of blood will be collected for this study on the day of the first study visit.

The extra blood samples required for this study will be collected during the same venepuncture as that usually undertaken on the day of receiving syphilis treatment. This would not cause any extra discomfort or additional risk of complications than that which may occur during routine venepuncture.

e) **e) Study sites**

Melbourne Sexual Health Centre (MSHC, Alfred Health) is the coordinating study site and will receive and analyse the data from all other study sites.

The other study sites have been selected as they are specialist sexual health services across Australia that see significant numbers of syphilis cases. These include the largest interstate sexual health clinics. This will allow for more rapid recruitment, particularly in women and heterosexual patient groups.

Sites involved include: Sydney Sexual Health Centre, Western Sydney Sexual Health Centre, the Gold Cost Sexual Health Service, the Tasmanian State-Wide Sexual Health Service, Adelaide Sexual Health Centre, Barwon Health (BRASH) Clinic and the Pacific Clinic (Newcastle).

Onsite recruiting clinical staff – nurses and doctors – at all sites, will be supported with training on the study procedures and sample collection.

f) **Recruitment**

Participants will be primarily recruited from MSHC. Other sites for sample collection include interstate sexual health clinics, see below. Consecutive patients at MSHC with a clinical or laboratory-confirmed diagnosis of secondary syphilis will be screened for eligibility either at triage, during consultation with the doctor/nurse or by the follow up nurse (FUN) recalling a client with positive syphilis results. The eligibility criteria for participants are able to be assessed as part of the routine questions asked in a sexual health clinic or as a consequence of positive syphilis testing results becoming available.

Once participants are approached for the study, they will be briefly informed about the nature of the study by a nurse or by the doctor during their consultation. During this process the consulting nurse or doctor will assess the understanding of the potential participant and their ability to consent. They will be offered a chance to participate in the study. If the participant wishes to take part in the study, they will be referred to the on-site research nurse or doctor.

Eligible participants at MSHC may be referred directly to the research nurse or obtain a booked appointment with a medical officer or research nurse.

Interstate sexual health clinics participating in their study will perform and collect their usual diagnostic samples as per their own in-house protocols. The additional collected study samples: saliva, oral swab, anal canal swab, vaginal swab, and blood sample will be transported to MSHC via Express post. No individually identifiable information will be included with these samples (only study ID, date of sample collection and participant date of birth on the study label). MSHC will then forward samples to VIDRL via existing courier pathways. Results of routine diagnostic swabs and blood tests from participants recruited interstate will be obtained later from the relevant clinic.

g) **Informed consent process**

Participants who are eligible will be provided with two copies of the Participant information and consent form (PICF) by the Research staff. The purpose and nature of the study will be explained in full in the PICF. Further information regarding the study will be available via a study phone line.

Potential participants will be asked to consent to the following, depending on their gender:

- Collection of additional saliva, oral swab, anal canal swab, blood sample, and vaginal swab (if indicated).
- Optional photography

The RN will be responsible for recruitment and explanation of the study to potential participants, obtaining informed consent, and explaining sample collection methods.

The MSHC result follow-up nurses (FUN) will also be responsible for advising potential participants of the study when they contact a client to recall them for treatment of new early syphilis.

Once verbal consent has been obtained the study investigator will inform the participant that they need to complete, sign and date the patient information and consent form.

h) **History**

A targeted medical history will be obtained, and will include:

- Current symptoms of syphilis, e.g., lesion, ulcer, pain, lumps, rash, neurological symptoms.
- Site and duration of symptoms.
- Previous history of symptoms of rash or lesions since the last syphilis test.
- History of antibiotic use or topical medication within the previous month.
- HIV status, antiretroviral therapy, and viral load
- Current HIV pre-exposure prophylaxis use
- Number of previous syphilis infections
- Any known contact of syphilis

i) **Clinical Examination overview**

- The SOS RN or SOS MO will conduct all basic study procedures.
- All participants must undergo MO review by the SOS MO or the on-duty clinical supervisor.
- Ensure all equipment and materials are prepared and all specimen containers labelled prior to the start of the examination
- A suggested examination flow would be:
- Oral cavity examination; and collection of oral swabs and/or saliva sample
- Anogenital examination and collection of anal/vaginal swabs
- Photography if indicated
- Phlebotomy
- Arrange for treatment
- Discuss how to access results.

j) **Treatment and follow up advice**

- The SOS MO or clinical supervisor is to provide participant with prescription for antibiotic treatment.
- Advise participant of when and how their routine results will be available.
- Advise participant that their individual study sample results will not be provided.
- All participants recruited for this study will receive antibiotic treatment for syphilis infection on the day of the study visit and be advised to notify all relevant sexual partners, to advise the partner(s) to also receive testing and treatment as a contact of syphilis infection.

k) **Follow up study procedures**

- Follow up collection of saliva, oral swabs, vaginal swabs, and anal canal swabs – Days 3, 7, 14, 30

l) **Reimbursement**

Participant reimbursement

There will be reimbursement of a $75 gift voucher or bank deposit provided for the first study visit. Those participants who attend for follow up visits will received an additional $50 for every follow up visit attended. Reimbursement is offered as compensation for the participant’s time, and is higher for the first study visit as this is longer than the follow up visits.

External site reimbursement

External sites will receive reimbursement for staff time involved in recruitment, performing the study visit and study administrative activities. External site reimbursement will be $100 for every participant recruited for a first study visit, and $75 for every participant follow up visit.

6. **PATHOLOGY TESTING**
a) **Overview**

Samples collected during the consultation may comprise routine STI testing as well as collection of the study samples. The microbiology specimens (by site) and laboratory test request for study sample collected will be recorded in an examination template.

b) **Study samples:**

All participants:

- Saliva, oral swab, anal canal swab, and blood.
- Vaginal swab, if indicated

Optional samples

- Photography

Study samples should be sent to VIDRL in a separate specimen bag to any routine STI diagnostic samples.

Study samples and accompanying request forms must be labelled with an identifying study sticker with study participant number, date of birth, site of sample and date of collection.

No further participant information is to be included on any study samples or the study request form.

c) **Routine STI testing**

STI screening will also be performed unless done within the last 14 days and the results are available at the time of the consultation.

All routine STI screening and testing is to be labelled, ordered through Clinical management practice software (CPMS), and submitted to the appropriate laboratory (VIDRL or Melbourne Diagnostic Unit, MDU) as per usual MSHC protocols.

d) **Transport**

All study samples from all recruitment sites, will be:

- couriered to VIDRL if the site has existing specimen transport pathways.

OR

- Sent via Express post if the site does not have existing specimen transport pathways.

e) **Storage of samples**

- All study samples sent to VIDRL will be stored frozen, regardless of the routine syphilis results. VIDRL will maintain a summary log of all results that links the study ID, the body site of the samples and the date of collection.
- Stored samples may be used for other research directly related to this study, and for *Tp* and WGS strain comparison.

7. **Results**
a) **Overview.**

Study results generated from all sites will be reported in two different ways: as routine results or study results. Routine results are those from STI testing performed on the same day as the study visit, including syphilis PCR results from mucocutaneous lesions, syphilis serology, and other STI results, as these are needed for timely management of syphilis infection and any other STIs.

Study results are those results from experimental study samples and includes *Tp PCR* results on blood, anal canal swab, vaginal swab, oral swabs, and saliva samples.

b) **Follow up of results**

MSHC: All routine results of STI screening, HIV, syphilis serology and *Tp* PCR results of swabs of mucocutaneous lesions will be reported back to MSHC from MDU and VIDRL via the usual reporting pathways. Any positive routine diagnostic or screening tests will be followed up as per usual existing follow-up nurse (FUN) processes. This represents the current standard of care for screening and testing for syphilis infection.

All results of study samples will be delivered directly to the study researchers and identified by the study number, type of sample and date of collection only.

c) **External results and information**

If the participant is recruited from a site other than MSHC, then the external site will retain responsibility for following up all routine syphilis and other STI results as per their usual protocols. Routine syphilis and other STI from participants recruited at external sites will be supplied to MSHC at a later date. The results of study samples collected at external sites will not be supplied to external recruitment sites, as they are non-validated samples that are not required for patient management.

d) **Communication of study results**

All participants recruited are selected as having either clinically suspected or laboratory-confirmed secondary syphilis and will receive antibiotic treatment for syphilis on the same day, at the conclusion of the study visit. All participants recruited will be advised on the day of the study visit to notify all relevant sexual partners and advise their partners to be tested and treated for syphilis infection.

The additional study samples collected in this study are not routinely used for diagnostic purposes. In fact, *Tp* PCR testing of blood, saliva, oral swabs, anal canal swabs and vaginal swabs, are not currently used for screening or for routine standard of care and are not validated diagnostic tests. The results of the study samples will not be communicated to the participant.

Aggregate results will be published in peer-reviewed journals. A plain language summary will be made available on the MSHC website and participants will be made aware of this in the Participant Information and Consent Form.

8. **Database collection, management and analysis**

Data on the characteristics of participants, syphilis testing, syphilis results and management will be extracted from routinely collected clinical information from the services electronic medical record either onto a paper-based proforma or into an MS Forms. The data from the MS Form will be automatically recorded in an Excel spreadsheet housed in a private MS Team accessible only to listed SOS2 researchers. Paper-based records, including PICFs, from external sites will be scanned and uploaded to Microsoft Teams, reviewed by the coordinating site (MSHC), who will then notify the external site to destroy the original paper-based document(s). Any data originating from uploaded paper-based documents will be added to the MS Form by MSHC research staff.

. The database will contain no name codes, dates of birth or UR numbers. All data will be stored in a potentially identifiable format, linked to a unique study identification number for each participant. The linking document will be separate from any health data. Participant information is protected and will only be used to facilitate the research. Information identifying an individual will not be disclosed to any third party. All data and photographs collected will be stored on a password protected institute server and only named investigators will have access to the data.

9. **Statistical considerations**
a) **Sample size and calculation**

The sample size for this study is based on the following analysis:

(1) 95% confidence intervals for possible prevalence rates for *TP* shedding from the oral or anal mucosa

**Table 1: Proportions of oral *T. pallidum* detected in previous studies**

| Author | Overall | PS | SS | ELS | LS | Sample |
| --- | --- | --- | --- | --- | --- | --- |
| Yang | 42.3 | 34.2 | 63.9 | 28 |  | Oral lesion or cavity |
| Golden | 37 | 50 | 50 | 11.1 |  | pharyngeal |
| Wang | 44.4 | 31 | 87.5 |  | 25.6 | saliva |
| Towns | 24 | 9 | 44 | 4 |  | Oral rinse/lesion |
| Tantalo | 27 | 14 | 40 | 4 |  | Oral cavity |
| Nieuwenberg | 30.8 | 10 | 64 | 24 |  | pharyngeal |

The proportion of oral *T. pallidum* detected from the oral cavity in previous studies is shown in table 1 above. 95% confidence intervals for possible proportions of men with *TP* shedding from the oral or anal mucosa are presented below. A total sample size of 50 would also give narrower 95% CIs than 25 but 100 does not reduce the CIs much further given the increase in sample size required, table 2 below.

**Table 2. Estimated 95% confidence intervals for proportions of individuals with oral, vaginal, and anal shedding and sample size**

| *TP* shedding (%) | 25 | 50 | 100 | 200 |
| --- | --- | --- | --- | --- |
| 5 | 0.0010-0.2035 | 0.0126-0.1655 | 0.0186-0.1183 | 0.0256-0.0927 |
| 10 | 0.0255-0.3122 | 0.0333-0.2181 | 0.0516-0.1804 | 0.0637-0.1523 |
| 20 | 0.0683-0.4070 | 0.1003-0.3372 | 0.1292-0.2943 | 0.1483-0.2636 |
| 30 | 0.1495-0.5350 | 0.1786-0.4461 | 0.2145-0.4011 | 0.2384-0.3694 |
| 40 | 0.2113-0.6133 | 0.2641-0.5482 | 0.3048-0.5030 | 0.3322-0.4717 |
| 50 | 0.3131-0.7220 | 0.3553-0.6447 | 0.3983 – 0.6017 | 0.4287-0.5713 |
| 60 | 0.3867-0.7887 | 0.4518-0.7359 | 0.4972-0.6967 | 0.5285-0.6685 |
| 70 | 0.5061-0.8793 | 0.5539-0.8214 | 0.6002-0.7876 | 0.6313-0.7626 |
| 80 | 0.5930-0.9317 | 0.6628-0.8997 | 0.7082-0.8733 | 0.7378-0.85312.5 |

b) **Analysis plan**

Statistical analyses of the study cohort will include, but will not be limited to:

- *Tp* PCR prevalence, viability, load, and duration of detection in samples collected in this study
- Comparison of *Tp* PCR load in oral swabs, saliva, anal canal swab, vaginal swab, and lesions to that in blood and each site of collection
- Load of *Tp* PCR in different samples and comparison with site of collection and patient subpopulation.
- Differences in above results according to HIV status, number of previous syphilis infections, gender.

10. **Confidentiality, storage, and archive of study documents**

Any electronic data will be password-protected and stored on a secure server that only named research personnel have access to. re-identifable data is

able to be stored on Alfred Health and Alfred Digital Health-approved Microsoft Office 365 platforms (MS Teams) with appropriate security settings

that confine access to named researchers only. These platforms have been tested and approved by Alfred Health’s data security department. These

platforms also disable sharing of data with unauthorized parties.

Each participant will be allocated a study ID number to allow coding of information.

Participant name, UR number and date of birth will be linked to study ID in a password protected Excel spreadsheet to enable re-identification. This spreadsheet will be stored on a secure server or other Alfred health approved platform (ie MS Teams) and only accessible to the research team.

All other study data will be stored against the study ID in a separate study database. This database will be stored on a secure server and only accessible to the research team. External sites will return required study information to the coordinating site via a private folder on an Alfred HREA approved platform (ie MS teams)

The PICF (and any other paper-based study documents) will be stored in a locked filing cabinet at the recruiting site that is only accessible to the researchers. External sites will be requested to scan the signed PICF and any other paper-based study documents and upload to a secure folder in Microsoft Teams once recruitment is complete. This will allow the coordinating site to ensure the form has been correctly completed and whether extended study participation has been consented to by participants.. They will also be able to scan the completed PICF and upload to the secure MS Teams platform.

Biological study samples containing the name of the study, study specific ID and the date of collection will be stored in freezers in a secure VIDRL department laboratory.

11. **Ethics**

The study protocol will be submitted for approval to the Alfred Hospital Ethics Committee.

12. **COVID-19 Contingency Plan**

This project will be carried out in accordance with any local COVID-19 restrictions and public health guidelines. Even in the strictest COVID-19 restrictions, it is still permissible for patients to leave home to access medical care as required. The first visit is a clinical visit to access treatment for syphilis infection. Recruitment into the study and collection of first visit samples and data poses no additional COVID19 risk to the patient or clinician due to their alignment with clinically required processes for syphilis management.

If a COVID-19-related lock-down or other travel restriction were to be imposed, due to local public health advice, then the additional extended visits (not clinically indicated for patient care)  would not be undertaken.

13. **References**

1.	Yang CJ, Chang SY, Wu BR, Yang SP, Liu WC, Wu PY, et al. Unexpectedly high prevalence of *Treponema pallidum* infection in the oral cavity of human immunodeficiency virus-infected patients with early syphilis who had engaged in unprotected sex practices. Clin Microbiol Infect. 2015;21(8):787 e1-7.

2.	Wang C, Hu Z, Zheng X, Ye M, Liao C, Shang M, et al. A new specimen for syphilis diagnosis: Evidence by high loads of *Treponema pallidum* DNA in saliva. Clin Infect Dis. 2020.

3.	Golden M, O'Donnell M, Lukehart S, Swenson P, Hovey P, Godornes C, et al. *Treponema pallidum* Nucleic Acid Amplification Testing To Augment Syphilis Screening among Men Who Have Sex with Men. J Clin Microbiol. 2019;57(8).

4.	Towns JM, Leslie DE, Denham I, Wigan R, Azzato F, Williamson DA, et al. *Treponema pallidum* detection in lesion and non-lesion sites in men who have sex with men with early syphilis: a prospective, cross-sectional study. Lancet Infect Dis. 2021;21(9):1324-31.

5.	Tantalo LC, Mendoza H, Katz DA, Sahi SK, Marra CM. Detection of *Treponema pallidum* DNA in oropharyngeal swabs and whole blood for syphilis diagnosis. Sex Transm Dis. 2021.

6.	Nieuwenburg SA, Zondag HCA, Bruisten SM, Jongen VW, Schim van der Loeff MF, van Dam AP, et al. Detection of *Treponema pallidum* DNA during early syphilis stages in peripheral blood, oropharynx, ano-rectum and urine as a proxy for transmissibility. Clin Infect Dis. 2022.

7.	Towns JM, Chow EPF, Wigan R, Fairley CK, Williamson D, Azzato F, et al. Anal and oral detection of *Treponema pallidum* in men who have sex with men with early syphilis infection. Sex Transm Infect. 2022.

8.	Phillips S, Vodstrcil LA, Huston WM, Lawerence A, Timms P, Chen MY, et al. Detection of *Chlamydia trachomatis* mRNA using digital PCR as a more accurate marker of viable organism. Eur J Clin Microbiol Infect Dis. 2018;37(11):2117-22.
